# Sparsely Wiring Connectivity in the Upper Beta Band Characterizes the Brains of Top Swimming Athletes

**DOI:** 10.3389/fpsyg.2021.661632

**Published:** 2021-07-16

**Authors:** Xinzhen Pei, Xiaoying Qi, Yuzhou Jiang, Xunzhang Shen, An-Li Wang, Yang Cao, Chenglin Zhou, Yuguo Yu

**Affiliations:** ^1^Human Phenome Institute, State Key Laboratory of Medical Neurobiology and Ministry of Education (MOE) Frontiers Center for Brain Science, School of Life Science and Research Institute of Intelligent Complex Systems, Fudan University, Shanghai, China; ^2^Shanghai Research Institute of Sports Science, Shanghai, China; ^3^Department of Psychiatry, Icahn School of Medicine at Mount Sinai, New York, NY, United States; ^4^School of Psychology, Shanghai University of Sport, Shanghai, China

**Keywords:** activity cost, energy efficiency, elite swimmers, phase lag index, wiring cost

## Abstract

Human brains are extremely energy costly in neural connections and activities. However, it is unknown what is the difference in the brain connectivity between top athletes with long-term professional trainings and age-matched controls. Here we ask whether long-term training can lower brain-wiring cost while have better performance. Since elite swimming requires athletes to move their arms and legs at different tempos in time with high coordination skills, we selected an eye-hand-foot complex reaction (CR) task to examine the relations between the task performance and the brain connections and activities, as well as to explore the energy cost-efficiency of top athletes. Twenty-one master-level professional swimmers and 23 age-matched non-professional swimmers as controls were recruited to perform the CR task with concurrent 8-channel EEG recordings. Reaction time and accuracy of the CR task were recorded. Topological network analysis of various frequency bands was performed using the phase lag index (PLI) technique to avoid volume conduction effects. The wiring number of connections and mean frequency were calculated to reflect the wiring and activity cost, respectively. Results showed that professional athletes demonstrated better eye-hand-foot coordination than controls when performing the CR task, indexing by faster reaction time and higher accuracy. Comparing to controls, athletes' brain demonstrated significantly less connections and weaker correlations in upper beta frequency band between the frontal and parietal regions, while demonstrated stronger connectivity in the low theta frequency band between sites of F3 and Cz/C4. Additionally, athletes showed highly stable and low eye-blinking rates across different reaction performance, while controls had high blinking frequency with high variance. Elite athletes' brain may be characterized with energy efficient sparsely wiring connections in support of superior motor performance and better cognitive performance in the eye-hand-foot complex reaction task.

## Introduction

Human brain is complex and has multiple levels of organization. The realization of cognitive function is a result of coordination and multilevel coupling of various brain regions, including information encoding, decoding, and communication (Jun et al., 2019). These processes come at high metabolic costs (Shulman et al., 2009) that are used for signaling activity (i.e., electrochemical signal generation, propagation, and synaptic communication across neurons; Herman et al., 2009; Sanganahalli et al., 2016; Yu et al., 2018) and for non-signaling processes (i.e., supporting housekeeping mechanisms and maintaining resting potential; Engl and Attwell, 2015; Yu et al., 2018). This is supported by experimental studies showing a large amount of energy are required to maintain the electrical activity of neurons and the organization of neural networks in the mammalian brain (Laughlin and Sejnowski, 2003; Hasenstaub et al., 2010; Sengupta et al., 2010). Previous study suggested that the high-order brain may make certain economic trade-offs during their function, tending to minimize the energy cost while maximize the output efficiency (Laughlin and Sejnowski, 2003). The energy consumption rate can be captured by electroencephalogram (EEG) frequency components and shows a linear relationship with the brain activity rate (Buzsaki et al., 2012). However, little is known about the determinants of the energy-efficiency in the brain. Recently, a study suggested that the learning process, which relied on synaptic plasticity, might promote efficient coding at a low cost (Yu et al., 2018). In the present study, we investigated whether the long-term professional athletic training such as swimming would influence the efficiency of energy consumption in the brain by alerting the functional connectivity.

In professional sports, the intrinsic functional state of the brain, such as the sensitivity of sensory perception, the degree of concentration, the speed of information processing, and the degree of neuromuscular control (Pei, 2020), is essential to athletes' performances. EEG is a non-invasive technology with a million second temporal resolution. It can be used to detect the neural activities from the scalp reflecting functional states of the brain. For example, golfers with expert putting skills showed increased frontal midline θ power and parietal α2 power (Baumeister et al., 2008), increased α and β power were found in the left hemisphere of rifle shooters during the preparation process before aiming, and increased θ power was found along the frontal midline during the aiming phase (Hillman et al., 2000; Doppelmayr et al., 2008); increased δ and θ frequency activity were found during ball sports exercises (Ermutlu et al., 2015); increased α activity was found in the left hemisphere of archers as the aimed (Salazar et al., 1990); increased α and β activity was recorded from widely distributed sites on the scalp after treadmill exercise (Mierau et al., 2009; Schneider et al., 2009); decreased α activity and increased β activity were found during cycling (Kubitz and Mott, 1996); and an increased α/β index in the frontal lobe was related to long-term fatigue from cycling (Nielsen et al., 2001).

Functional connectivity is used to quantify statistical interdependencies among physiological time series recorded from different brain areas (Lee et al., 2003; Fingelkurts et al., 2005). The brain functional connectivity can be evaluated by coherence, Granger causality (Granger, 1969), phase coherence (Tass et al., 1998), synchronization likelihood (Stam and van Dijk, 2002), phase lag index (PLI) (Stam et al., 2007), and the imaginary part of coherency (Nolte et al., 2004). PLI quantifies connectivity strength on the basis of phase synchronization and was designed to overcome the volume conduction problem (Stam et al., 2007). Research has found that an individual's functional brain connectivity profile is unique and similar to one's fingerprint (Finn et al., 2015). An individual can be identified from a large group of subjects solely relying on the basis of the connectivity matrix, especially in the frontoparietal networks (Finn et al., 2015). In the context of sports, distinguish relationships between different sport events and the characteristics of brain networks have been reported. For example, functional connective edges in the right hemisphere was significantly greater than those in the left hemisphere during shooting (Liwei et al., 2018). Table tennis players showed reduced EEG coherence in multiple frequency bands comparing to novices (Zhiping et al., 2016). However, there has scarce EEG research on the brain functional connectivity to reflect the cost-efficiency of professional athletes.

Swimming is a speed event relying on cyclical movements and requires high levels of reaction, movement and displacement speeds. Long-term systematic physical and skill training leads to superior reaction behaviors in professional athletes (Mori et al., 2002; Williams et al., 2002; Kida et al., 2005; Simonek, 2011). Specifically, a study showed that the hand-foot coordination was positively correlated with swimming speed and competitive performance (Takagi et al., 2004). In the same vein, the complex reaction (CR) (i.e., a type of choice reaction) is considered as a behavioral characteristic that distinguishes elite swimmers from thousands of beginner swimmers (Guang et al., 2013). However, it is unknown what role energy cost-efficiency plays in the CR performance. Hence, the present study aimed to (1) seek for potential electrophysiological markers identifying top swimmers; and (2) explore the brain energy cost-efficiency of ES using functional connectivity methods.

## Materials and Methods

### Participants

Twenty-one elite swimmers (ES) from Shanghai Swimming Management Center (Supplementary Tables 1–4) and 23 college students with no history of specialized swimming or other professional sports training (control group, CG) were recruited. All participants read and signed informed consent forms. The study was approved by the Ethics Committee of the Fudan University. Participants were all right-handed. The handedness was determined by self-reports and verified by the observation of their hand use in writing and performing the task. None of them had reported a history of mental illness. The EEG and behavioral data of 5 elite swimmers and 7 controls were excluded from the analysis because their EEG signals contained too many instabilities or drifts. The final sample consisted of 16 ES and 16 CG (*n* = 32, 15 Female). Sixteen swimmers (13 master-level) were distributed to 5 specialties (6 Free style; 4 Backstroke; 3 Breaststroke; 2 Individual Medley; 1 Butterfly) (for participant demographics, see Supplementary Tables 1, 2).

### Experimental Procedures

**Figure 1:**
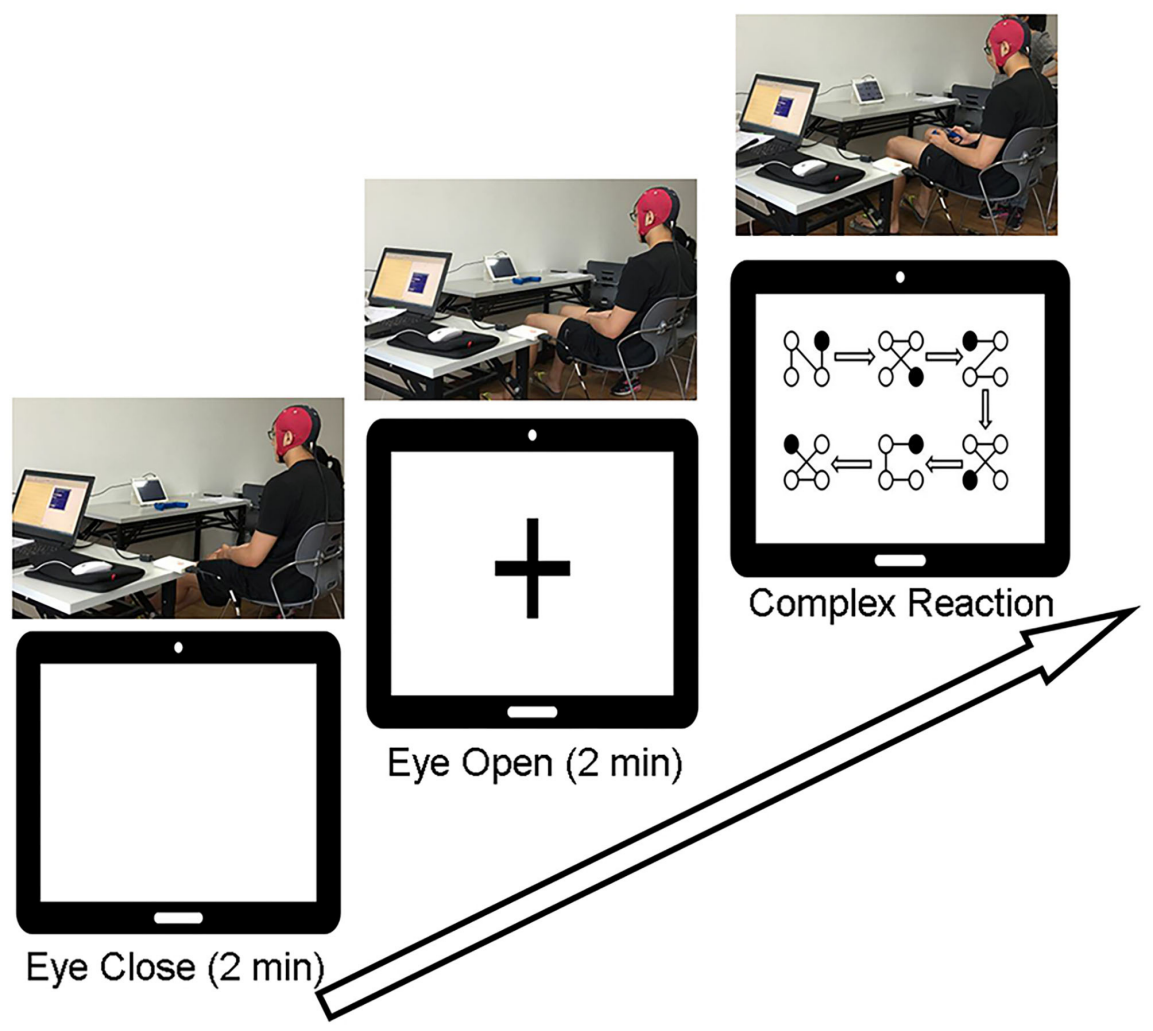
Experimental procedures. Participants were asked to keep their eyes closed for 2 min without thinking about anything and then to keep their eyes open and fixed on the “+” in the middle of the screen for 2 min. After a 1-min break, they were required to complete 8 trials of the complex reaction task according to the instructions on the screen.

Participants were seated comfortably in an armchair in a soundproof room. The experiment started with a 2-min waking eyes-closed (EC) period, followed by a 2-min eyes-open (EO) period with eyes fixed on a screen with a crosshair. Then, participants were required to perform the CR task while keeping the head still, followed by another EC (2 min) and EO (2 min) states. After each state, there was a 1-min short break. EEG signals were recorded throughout the process. Prior to perform the CR task, participants were instructed to practice several trails till they successfully completed one trail by themselves. In the CR task there were 8 trails and each trial involved 6 different blocks connected by directional arrows, and every block contained 4 balls connected by lines. The participant was required to use left/right finger or foot to indicate the location of the ball which appeared in one of the four corners of the screen (i.e., left finger = upper left, right finger = upper right, left foot = lower left, and right foot = lower right). If the participant responded correctly, the ball would disappear. Participants were required to respond as quickly and accurately as possible while keeping the head stable. The trial-by-trial reaction time and the total number of errors were documented. The experimental procedure is illustrated in detail in Figure 1.

### EEG Recording and Data Preprocessing

EEG signals were recorded from an 8-channel EEG system (eego, ANT Neuro, Berlin, Germany) and digitized at a sampling rate of 1,000 Hz. The reference electrode was placed between the Cz and Pz channels, and others (Fpz, Fz, F3, F4, Cz, C3, C4, and Pz) were distributed around the frontal and parietal areas according to the extended 10–20 international system. The impedance of all electrodes was kept below 10 kΩ.

EEG data were preprocessed in EEGLAB v.13.0.0.b, a MATLAB-based open toolbox (Delorme and Makeig, 2004). Segments with a duration of 1 min (from 30 to 90 s of the collected 120 s data) were selected from the EC and EO resting-state EEG, respectively, and all data associated with the CR task were imported for preprocessing. Raw data were re-referenced to the common average reference and filtered to a frequency range of 0.5–30 Hz. After running eye blink recognition, artifacts associated with eye movements and blinks were removed by using the AAR1.3 toolbox plugin, which performed automatic electrooculogram (EOG) artifact correction using blind source separation (BSS) and identified the EOG components using fractal analysis (Gomez-Herrero et al., 2006). After that, the resting states and CR task data were all segmented into epochs with a duration of 2 s each. Subsequently, the epochs with abnormal values beyond the upper limit of 75 μV were rejected (Collin et al., 2012). Overall, 10.1% epochs from the resting states and 16.2% from the CR task were excluded due to artifact contaminations.

It's well-known that the alpha blocking phenomenon appears during relax wakefulness and conceptualized as desynchronized neural population activity during active stimuli. Previous study has demonstrated that the alpha blocking phenomena could reflect wakefulness-to-sleepiness levels (Jiao and Lu, 2017). They estimated the degree of falling in sleepiness from drivers' wakefulness by calculating the alpha blocking rate from EEG wave. In present study, we calculated the alpha blocking rate (α_blockingrate_) to reflect a subject's switch from eye-close (EC) to eye-open (EO) states as an estimate of degree of wakefulness as well as stability of subjects during experiment. We used the rate of change of the alpha blocking rate (α_blockingrate_) effect of the EC and EO resting states before and after the CR task to monitor the stability of the experimental recording process and verify the validity of EEG data after preprocessing (Zheng et al., 2018). We set 20% as the stability threshold of the rate of change of the alpha blocking rate based on our long-term observations in experimental study. We observed that once subject's α_blockingrate_ decreased its value above 20% change, it was very likely that the subject became sleepy in long-term experiment. On the contrary, if it increased its value above 20% change, it was very likely the intrinsic brain behavior state had changed, which introduced some unexpected noise to the experiment, and affected the interpretability of the data. After the CR task, the α_blockingrate_ values of the 2 groups were both reduced by <20% in comparison to those before the CR task (Table 1, Supplementary Figure 1, and Supplementary Method 1).

**Table 1:** Descriptive statistics for the variables of interest.

	**ES**	**CG**	
	**Mean ±*SD***	**Mean ±*SD***	***P*-value**
**Complex Reaction (CR)**			
CR reaction time	15.85 ± 4.19 s^*^	19.06 ± 5.13 s^*^	0.033
CR accuracy	94.86% ± 2.51%^*^	87.79% ± 8.99%^*^	0.005
CR speed	4.08 ± 0.8 trials/min^*^	3.47 ± 0.92 trials/min^*^	0.049
**^α^blocking rate**			
Before CR tasks	78.55% ± 22.83%	71.92% ± 16.71%	0.356
After CR tasks	73.54% ± 26.2%	61.85% ± 20.2%	0.168
**Lateralization Index (LI)**			
EC LI_frontal_	−0.011 ± 0.156	−0.050 ± 0.198	0.552
EC LI_parietal_	−0.092 ± 0.225	0.009 ± 0.212	0.200
EO LI_frontal_	0.008 ± 0.150	−0.066 ± 0.117	0.128
EO LI_parietal_	0.029 ± 0.203	−0.048 ± 0.181	0.269
CR LI_frontal_	0.058 ± 0.169	0.012 ± 0.095	0.351
CR LI_parietal_	0.027 ± 0.080^*^	−0.069 ± 0.118^*^	0.012
**Wiring Connections (W**_**NC**_**)**			
1–4 Hz	6.31 ± 3.54	5.69 ± 3.11	0.600
4–8 Hz	13.00 ± 5.74	10.31 ± 4.42	0.148
8–13 Hz	9.38 ± 3.69	7.69 ± 3.99	0.224
13–20 Hz	8.38 ± 4.43	8.63 ± 4.37	0.873
20–30 Hz	6.38 ± 4.73^*^	9.88 ± 4.47^*^	0.040

* *p < 0.05, significantly different between elite swimmers and the control group*.

### Network Wiring Connections Based on Phase Lag Index

**Figure 2:**
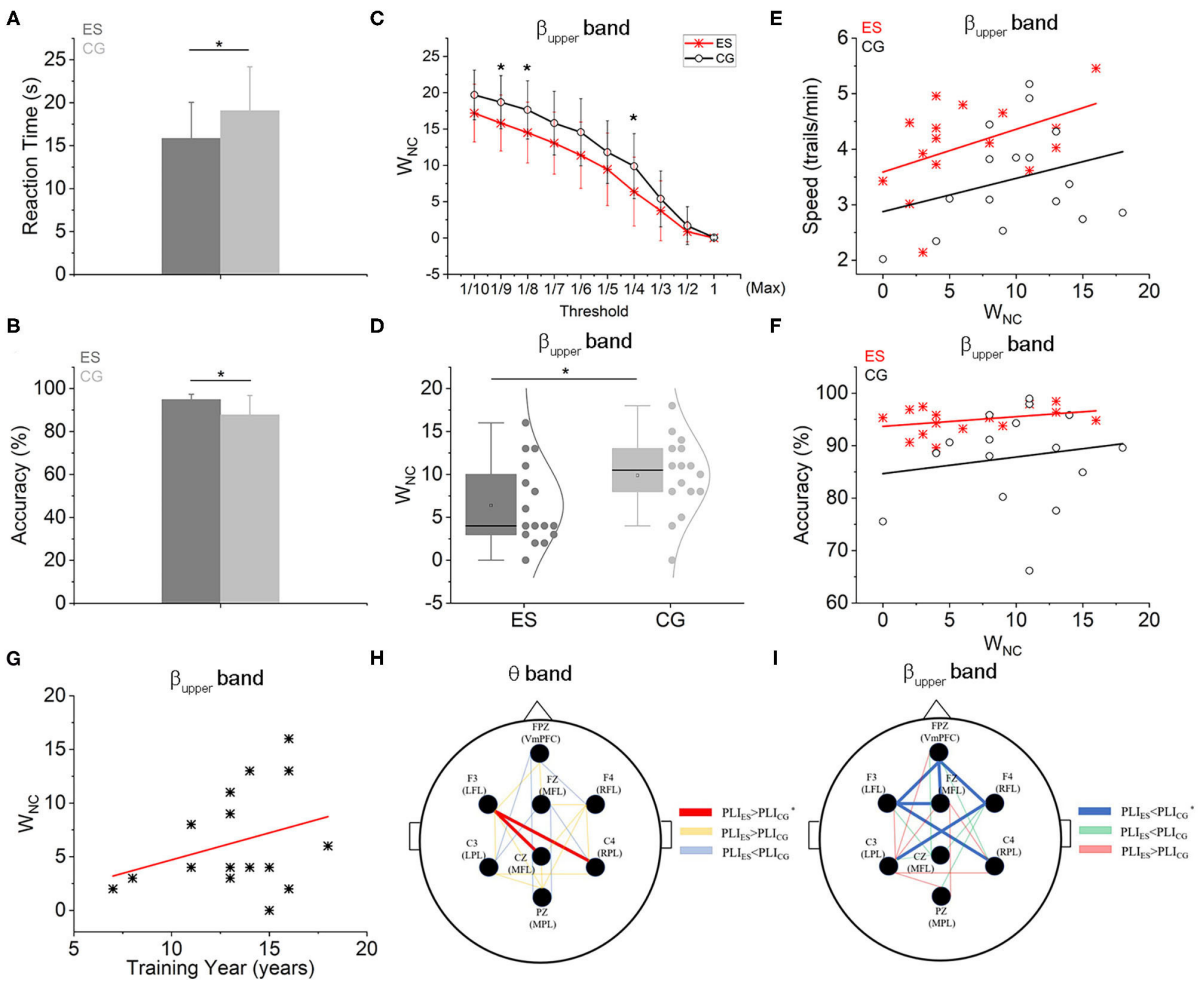
Functional connectivity and network wiring cost-efficiency characteristics based on PLI. **(A)** The CR responding time of ES was significantly shorter than controls. **(B)** The CR accuracy of ES was also significantly higher than CG. **(C)** The relationship between WNC of two groups and different threshold settings. When the threshold was set as the 1/4Max, 1/8Max, and 1/9Max, the WNC of ES was significantly less than that of CG in the upper beta frequency band. **(D)** The W_NC_ of ES was significantly less than controls in the upper beta frequency band when the threshold was set as the 1/4 Max. **(E)** Both in the ES and CG groups, W_NC_ was positively correlated with the CR speed (S) in the upper beta frequency band. But in the ES, the intercept was greater [S_ES_ = 3.588 + 0.077 × W_NC_, *r*(16) = 0.458; S_CG_ = 2.876 + 0.06 × W_NC_, *r*(16) = 0.292]. **(F)** There were positive linear relations between W_NC_ and CR accuracy (AC) both in the ES and CG group in the upper beta frequency band [AC_ES_ = 93.677 + 0.185 × W_NC_, *r*(16) = 0.349; AC_CG_ = 84.668 + 0.316 × W_NC_, *r*(16) = 0.157]. **(G)** In the ES group, there was a positive linear relation between W_NC_ and swimming training year (T) in the upper beta frequency band [W_NC_ = −0.323 + 0.503 × T, *r*(16) = 0.312]. **(H)** In the θ frequency band, there were 2 SCEs between ES and CG, connecting F3 to Cz and C4 (red lines indicate that the PLI values of ES were significantly higher than those of CG in the SCE topologic map, yellow lines indicate that the PLI values of ES were higher than CG but no significance, blue lines indicate the PLI values of ES were lower than CG but no significance). **(I)** Six SCEs were shown at the upper β frequency band between the 2 groups: Fpz-Fz, Fpz-F3, Fpz-F4, Fz-F3, F4-C3, and F3-C4 (blue lines represent that the PLI values of ES were significantly lower than those of CG in the topological map of SCEs, green lines represent the PLI values of ES were lower than CG but no significance, red lines represent the PLI values of ES were higher than CG but no significance). *p < 0.05, compared with the control group.

EEG network connectivity such as functional connectivity generally refers to the statistical relationship of EEG signals between electrodes (or brain areas) (Fingelkurts et al., 2005). To avoid the effect of volume conduction and the field diffusion on multiple-recording channels, we used the phase lag index (PLI) based on phase synchronization to evaluate the brain EEG functional connectivity (Stam et al., 2007). The PLI value was calculated with the open source toolbox HERMES based on MATLAB (Niso et al., 2013). The range of PLI values is generally between 0 and 1, where a value of 1 means that the 2 EEG signals have strict phase locking at a constant non-zero phase lag and a value of 0 means no coupling (or coupling with a relative phase that encircles 0 modπ, which is likely to result from volume conduction) (Zheng et al., 2018). Thus, the larger the PLI value indicates the stronger the non-zero phase synchronization and the stronger the connectivity (Stam et al., 2007). We first applied an approach called network-based statistics (NBS) (Zalesky et al., 2010) to analysis brain functional connectivity based on PLI and to find the significant connectivity edge (SCE). In terms of the energy related to the wiring cost. The weaker or less extensive the connectivity is, the less active synaptic connections there are. The less active synaptic connections will cost less energy. That is, network wiring cost (C_w_) is proportional to the wiring number of connections (W_NC_) (Achard and Bullmore, 2007; Zheng et al., 2021). There were up to 28 edges among the 8 electrodes. Some PLI values were very low which were around noise level. There should be an optimal baseline and threshold to reduce the noise interference. Hence, we set different thresholds e.g., from 1/10, 1/9, 1/8, 1/7, 1/6, 1/5, 1/4, 1/3, 1/2–1 maximum of PLI value of 32 participants (Max) in order to compare those relatively stronger functional connections for both ES and CG in the CR task state. There was a significance between 2 groups for some threshold (1/4 Max, 1/8 Max, and 1/9 Max) (Figure 2C and Supplementary Figure 10). Here, as an example case, $\frac{1}{4}$ Max was used as the threshold for distinguishing the talented swimmer group from the control. Absolute threshold was better than the relative threshold of each subject for the comparability between ES and CG. Therefore, we set 1/4 of the maximum PLI value of 32 participants in 2 groups as the threshold in each frequency band. If the PLI of two channels was greater than the threshold value, one W_NC_ was calculated. The W_NC_ of all participants were calculated and analyzed during the CR task.

### Activity Cost Based on Mean Frequency

Brain signaling activity involves not only a wiring cost for network connectivity but also an activity cost for neuronal discharge. Studies have shown that a higher mean frequency (MF) of EEG reflected higher levels of cerebral blood flow and metabolism (Ingvar, 1971; Hyder et al., 2013), and MF was confirmed to have a positive correlation with these physiological variables (Ingvar et al., 1976; Zheng et al., 2021). That is, the high MF suggests higher frequency of brain electronic activity that will cost more metabolic energy (Hyder et al., 2013; Yu et al., 2018). Since the MF can indirectly reflect the energy cost of neural electrical activity in the brain, we used the MF of the CR task state as the index for the energy cost of brain activity (C_activity_). MF will be higher or lower when the condition is changed. In the present study, we calculated and compared the MF of ES and CG groups at the same condition.

Calculation of MF: Each subject was selected 50 s signals of EC state, 50 s signals of EO state, and 70 s signals of CR task (selection from the beginning recording signals of the CR task) to be preprocessed. The mean frequency of 25 segments or 35 segments EEG data from 8 channels were calculated in 2 s. It is known that electromyogram (EMG) artifacts have a higher amplitude than the EEG signals and can be removed by using independent component analysis (ICA) technique (Chen et al., 2014; Frolich and Dowding, 2018). But in our present study, there were only 8 electrodes and they were not enough for using ICA to detect the EMG artifacts. According to the characteristics of EMG, such as distributing relatively higher frequency with a higher amplitude, we used the ratio in the formula (1) for the evaluation of the muscular content. The electromyography (EMG) artifacts were removed according to the ratio of high-frequency bands power over low-frequency bands power due to the relatively higher frequency of myoelectric. The calculation formula of the ratio was as follows:

(1) $$\text{Ratio }=\frac{\sum_{i\;=\;13}^{30}P(f_{i})}{\sum_{i\;=\;1}^{13}P(f_{i})}$$

Where *i* represents an integer frequency ranging from 1 to 30 Hz, and *P(fi)* means the power value at a certain integer frequency. When the value of Ratio of one segment from one channel of a subject was >1, this segment was removed because it belongs very likely to the EMG artifacts (Supplementary Figure 14 and Supplementary Method 4). According to the above methods, about 2.81‰ segments of EC, 8.59‰ segments of EO, and 7.03‰ segments of the CR task were removed before calculating the mean frequency of each channel of every participant in 3 conditions (EO, EC, and the CR task).

### Spectrum Power and Activation Rate

Averaged power spectra were computed across segments of different states in each participant. The power values were calculated for 5 frequency bands (δ: 1–4 Hz; θ: 4–8 Hz; α: 8–13 Hz; β_lower_: 13–20 Hz; β_upper_: 20–30 Hz). It has been well-established that low-frequency signaling activities, such as δ or θ, are related to sleep or the resting state of the brain, while high-frequency signaling activities, such as α and β, are related to the cognitive function (Kumar and Bhuvaneswari, 2012). The power ratio of the high-frequency band to the low-frequency band can reflect the degree of brain activation (Cheron et al., 2016). Therefore, we calculated the power ratio of the upper β frequency band to the θ frequency band of each channel (upper beta/theta ratio, or UBTR), to represent the activation rate of eight brain areas (Arns et al., 2013; Vollebregt et al., 2015). The formula of UBTR was as follows:

(2) $$\text{UBTR }(\%)=\frac{Power_{upper\;beta}}{Power_{theta}}\times 100$$

Note: In our study, there were no electrodes in the temporal or occipital areas of the brain. When we drew the spectrum power and mean frequency topologic maps, we added an additional 18 electrodes and set the values to 0 to avoid the influence of the frontal and parietal signals on the periphery. The added electrodes were Fp1, Fp2, F5, F6, C5, C6, P5, P6, Po3, Po4, F7, F8, O1, O2, T7, T8, Po1, and Po2.

### Blink Recognition

The electrical potential of eye blinks is required along with the brain rhythm signals by the electrodes in EEG and shows a higher intensity in the frontal electrodes and possesses higher amplitude than the brain rhythms (Sovierzoski et al., 2008; Al-gawwam and Benaissa, 2018). In present study, blink recognition was performed based on the EEG data of each participant after high- and low-pass filtering. The processes of algorithm identification were: (1) to find all the peaks that may be blinks in the Fpz channel using the function *findpeak* in Matlab (seen Supplementary Method 3); (2) to select 250 ms time series before and after each peak and calculate the amplitude of the peak to the trough on the left and right sides, respectively, and then to compare the averaged left and right amplitudes with the peak threshold. It would be kept when the averaged amplitude was larger than the peak threshold; (3) to meet the condition that there were at least in 3 other channels the amplitudes of the trough were greater than the trough threshold at the same period; (4) to remove the local maximum peak with the amplitude difference between 2 consecutive peaks less than one third of the maximum peak in the Fpz channel. The peak threshold was set to 2/5 of the maximum peak amplitude of the subject and the trough threshold was set to one third of the lowest trough amplitude of the subject in the Fpz channel during the complex reaction task. One blink was recognized after the above 4 conditions were all met.

According to the above-mentioned blink recognition algorithm, all the blinks of each subject were identified during the complex reaction task. The peaks of blinks were aligned to 0 ms. The mean amplitude from 250 to 150 ms before the blink peak was set as the baseline amplitude. Task-evoked blink potentials of each subjects were plotted after normalized by the Z-score method.

Blink rate or the frequency at which the eyelids open and close has been proposed and used to study cognitive control, learning, working memory, and decision making (Eckstein et al., 2017). In the manuscript, the instantaneous blink rate of each subject was calculated over time from the beginning to 60 ms of the complex reaction task, and was normalized with the average blink frequency of his/her group in 60 s. In the calculation process, subjects who blinked <3 times in the first 60 s should be removed.

Previous study noticed that the interblink intervals were quite variable between subjects (Ponder and Kennedy, 1928). According to the percentages of different blink intervals to the total number of blink intervals of each participant, the interblink interval histogram (IBIH) of each person in the process of CR tasks was calculated for his/her total CR tasks continuous time sequence s selected from each subject in the 2 groups. The total number of blink intervals of all subjects in each group was calculated with the time bin of 1 s. Most interblink interval durations of these subjects were distributed in <20 s while a very few interval durations (>20 s) distributed sparsely with maximal value reaching 91 s.

### Statistical Analysis

Brain functional connectivity based on PLI was analyzed mainly by an approach called network-based statistics using the NBS v1.2 toolbox, based on MATLAB (Zalesky et al., 2010). After 5,000 permutation tests, if there was a significant difference (*p* < 0.05) between ES and CG groups, it was marked with a line as a SCE in the topology diagram (Figures 2H,I). Other index data were analyzed by SPSS 20.0. An independent *t*-test was used between the 2 groups in one state such as the speed and accuracy of the CR task, WNC at different frequency bands or different threshold, the lateralization index in the resting or task state, mean frequency in the resting or task state, spectrum power at different frequency bands etc. A paired *t*-test was used between 2 states within the same group, e.g., mean frequency of ES between EC and EO state or between EO and the CR task state; the significance threshold was set at *p* < 0.05. Pearson correlation analysis was used for individual EEG and CR task performance.

## Results

### Complex Reaction Task Performance

The averaged reaction time and accuracy values of the CR task were shown in Table 1. The ES responded significantly faster (Figure 2A) and more accurate (Figure 2B) than the CG. This result is consistent with reports from other sports (Mori et al., 2002; Williams et al., 2002; Kida et al., 2005).

### Alpha Blocking Rate

Alpha activity is greatly reduced by the increase in light input from the resting EC state to the EO state or blocked by other attention-related signals. The alpha-blocking phenomenon is conceptualized as desynchronized neural population activity during active stimuli, and alpha blocking rate (α_blockingrate)_ between the EC and EO states is used to monitor the stability of the experimental recording process (Method seen Supplementary Method 1). It is generally believed that a data recording process is relatively stable if the α_blockingrate_ is <20% before and after the tasks (Bazanova and Vernon, 2014). In the current study, the mean α_blockingrate_ of the elite swimmers after the CR tasks was 6.4% lower than the rate before the tasks. In the control group, the α_blockingrate_ after the CR task was reduced about 15% (Table 1 and Supplementary Figure 1). This relatively higher and stable α_blockingrate_ values in elite athletes might reflect that they could maintain sustained attention for relatively longer period than controls.

### Network Wiring Connections

Based on network-based statistical analysis, there were 2 strengthened functional connectivity edges in the θ frequency band (4–8 Hz) in the ES compared with the CG during the CR task (Figure 2G). These edges were distributed in the left frontal area, connecting the left frontal area (F3) to the central parietal area (Cz) and to the right side of the parietal area (C4). In the upper β frequency band (20–30 Hz), compared to the college student controls, master swimmers had 6 less correlated functional connectivity edges (Figure 2H), which were mainly distributed in the frontal, temporal and parietal regions of the brain. The connection strengths of all recording sites for other frequency bands were not significantly different between the 2 groups.

In the upper β frequency band, the frontoparietal network W_NC_ of ES was significantly lower than that of CG (*p* < 0.05; Figure 2D) and was positively correlated with swimming training years within the ES group (Figure 2G). As shown in the Supplementary Figure 2, there were positive linear relations between training years and CR accuracy or speed. However, in the other 4 frequency bands, W_NC_ was not different between ES and CG (Supplementary Figure 3). Within the group either ES or CG, W_NC_ was also positively correlated with CR speed (Figure 2E) or accuracy (Figure 2F). However, the 2 intercepts of linear relations of ES were both greater than controls.

### Activity Cost Based on Mean Activity Frequency

**Figure 3:**
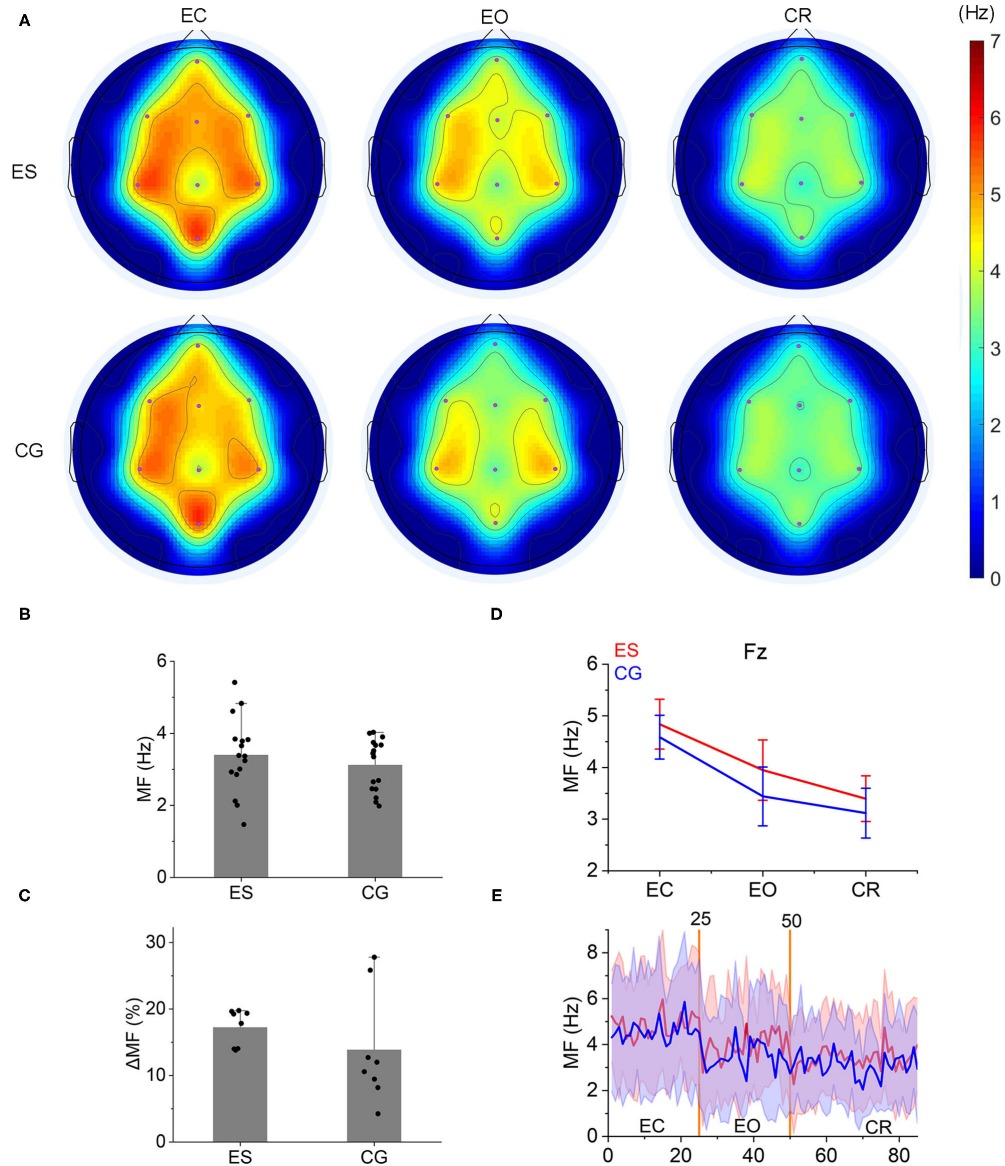
Mean frequency topology and characteristics in the 2 groups. **(A)** From the EC state to the EO state and then to the CR task state, a gradual decrease was shown in the MF topology both in ES and CG. **(B)** During the CR task, the MF of ES was slightly higher than that of CG, but there were no significant differences between the 2 groups (*P* > 0.05). **(C)** The change rate of the MF [(MF_eo_ – MF_cr_)/MF_eo_ × 100] from the EO condition to the CR task did not show significant differences between ES and CG. **(D)** The MF of the 2 groups in the Fz channel decreased from the resting state (EC and EO condition) to the CR task state. **(E)** The change trend of MF at the Fz channel from the resting state to the CR task state.

A gradual decrease was shown in the mean frequency of the frontoparietal area from the EC state to the EO state and then to the CR task (Figures 3A,D,E and Supplementary Figure 4). This result was consistent with our group's previous findings on the mean frequency of the normal population in the frontoparietal area (Supplementary Figure 5). However, during the CR task, there was no difference in the mean frequencies between ES and CG (*p* > 0.05; Figure 3B), nor was the change rate of the mean frequency from the EO state to the CR task (*p* > 0.05; Figure 3C).

### Spectrum Power Analysis and Activation Rate

Although the absolute power values of ES in the 5 frequency bands were not significantly different from those of CG during the CR task (*p* > 0.05), there was an upward trend in the ES in the frontoparietal region of the left hemisphere while it was absent in the CG (Supplementary Figure 6).

**Figure 4:**
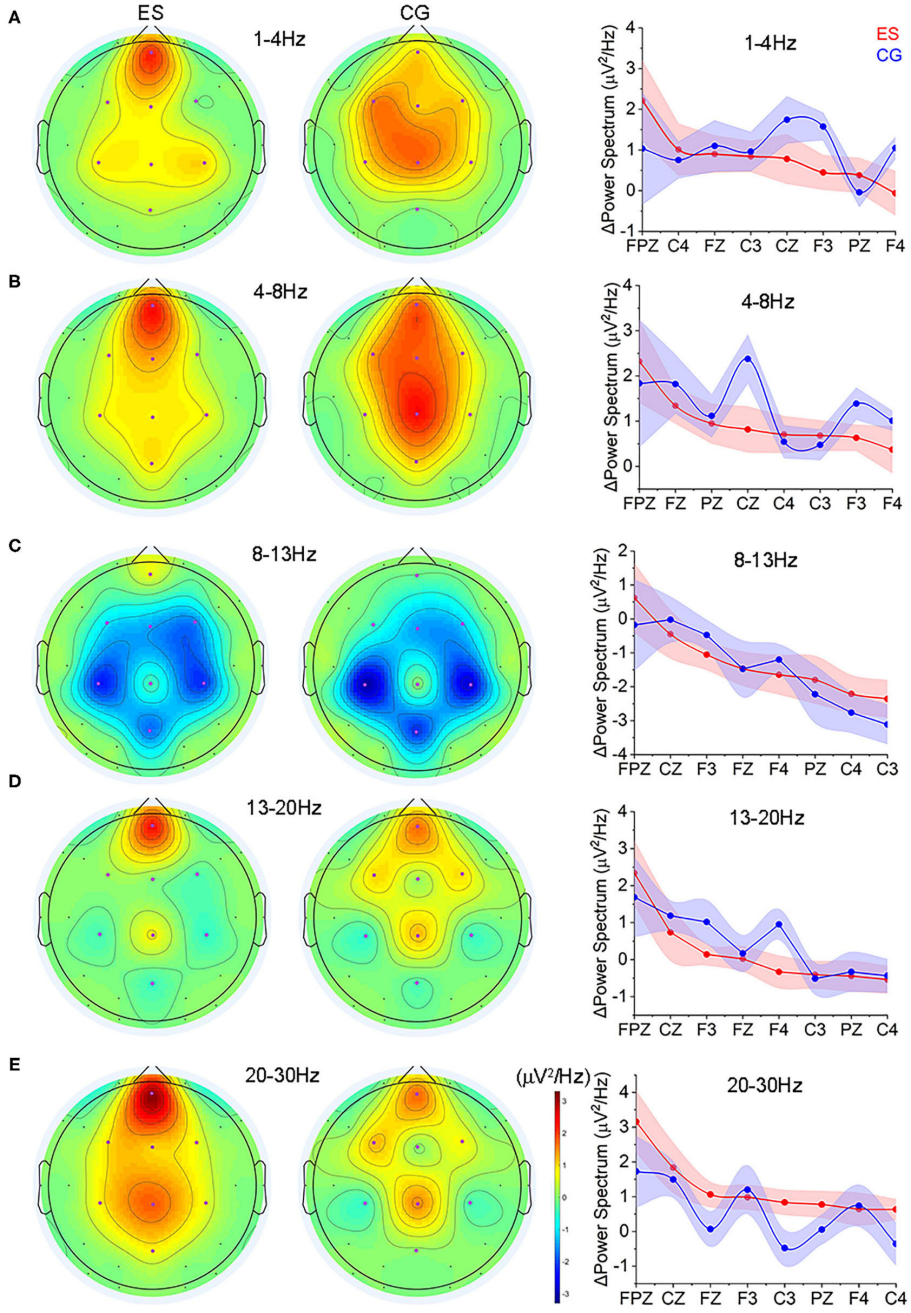
The changes of spectrum power from the resting EO condition to the CR task state. **(A)** Topology of the power changes in the frontoparietal region at the δ (1–4 Hz) frequency band and the differences in the change in each channel at this frequency band between ES and CG. **(B)** Topology of the power changes in the frontoparietal region at the θ (4–8 Hz) frequency band and the differences in the change in each channel at this frequency band between the 2 groups. **(C)** Topology of the power changes in the frontoparietal region at the α (8–13 Hz) frequency band and the differences in the change in each channel at this frequency band between the 2 groups. **(D)** Topology of the power changes in the frontoparietal region at the lower β (13–20 Hz) frequency band and the differences in the change in each channel at this frequency band between the 2 groups. **(E)** Topology of the power changes in the frontoparietal region at the upper β (20–30 Hz) frequency band and the differences in the change in each channel at this frequency band between the 2 groups.

Comparing to the EO state, ES showed increased activities in the prefrontal region at each frequency band during the CR task state, while CG showed more spatially distributed across multiple regions (Figures 4A–E and Supplementary Figures 6, 7). In terms of the absolute value of the power change, at the δ frequency band (1–4 Hz), ES had significantly smaller power changes in the left frontal area (F3) than CG (Figure 4A); in the θ frequency band (4–8 Hz), there was a significant difference in the center of the parietal area (Cz), and the power change of the ES was significantly smaller than that of the CG (Figure 4B); at the lower β frequency band (13–20 Hz), ES had a significantly smaller change in the right frontal area (F4) (Figure 4D); and in the upper β frequency band (20–30 Hz), an increased power change was found in the left parietal lobe (C3) of ES (Figure 4E).

**Figure 5:**
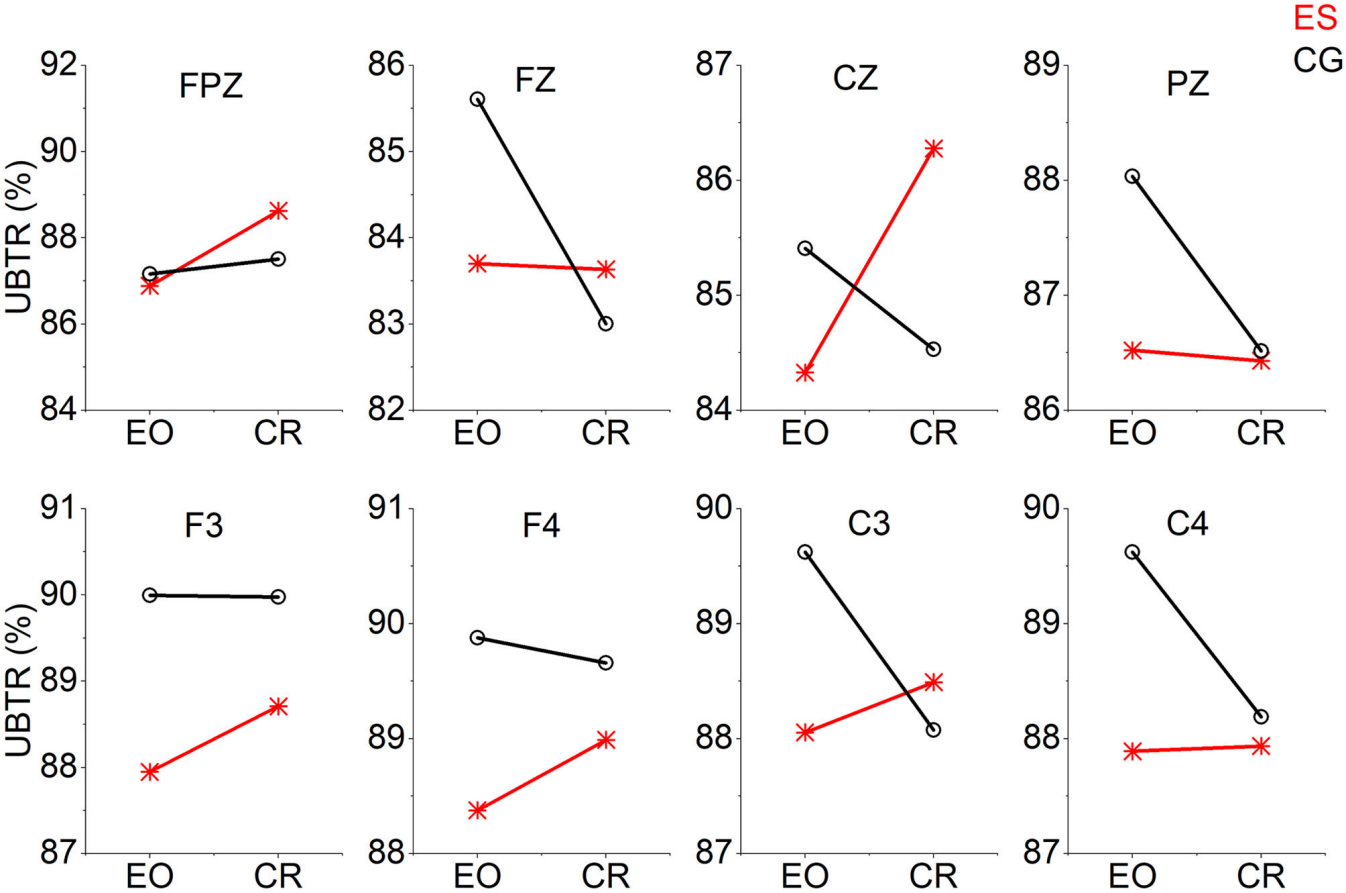
The trend graph of UBTR changes at 8 channels from the EO condition to the CR task condition in the 2 groups. The UBTR changes of the 2 groups interacted with 5 channels (F3, F4, Cz, C3, and C4).

From the EO state to the CR task state, the UBTR of ES decreased significantly less than that of CG in the frontal Fz channel (Figure 5). There were 5 channels (F3, F4, Cz, C3, and C4) in which the UBTR changes of the 2 groups interacted. That is, there was an increasing trend in the UBTR change of the ES, while there was a decreasing trend in that of the CG. However, only in the Cz channel was there a significant difference (Figure 5).

Supplementary Table 2 and Supplementary Figure 8A showed the frontal lateralization index (LI_f_) (Method seen Supplementary Method 2). There were no significant differences between ES and CG either in the resting state (EC and EO) or in the CR task state (*p* < 0.05). However, the parietal lateralization index (LI_p_) of ES was significantly different from that of CG in the CR task state (*p* < 0.05). During the CR task, the LIp value of ES was positive and close to zero, while that of CG was negative and far from 0. This result suggested that top swimming athletes had more balanced left and right cerebral hemisphere activities and more strengthened activation in the right hemisphere than college students (Table 1 and Supplementary Figure 8B).

### Blinks and Burst Blinks

**Figure 6:**
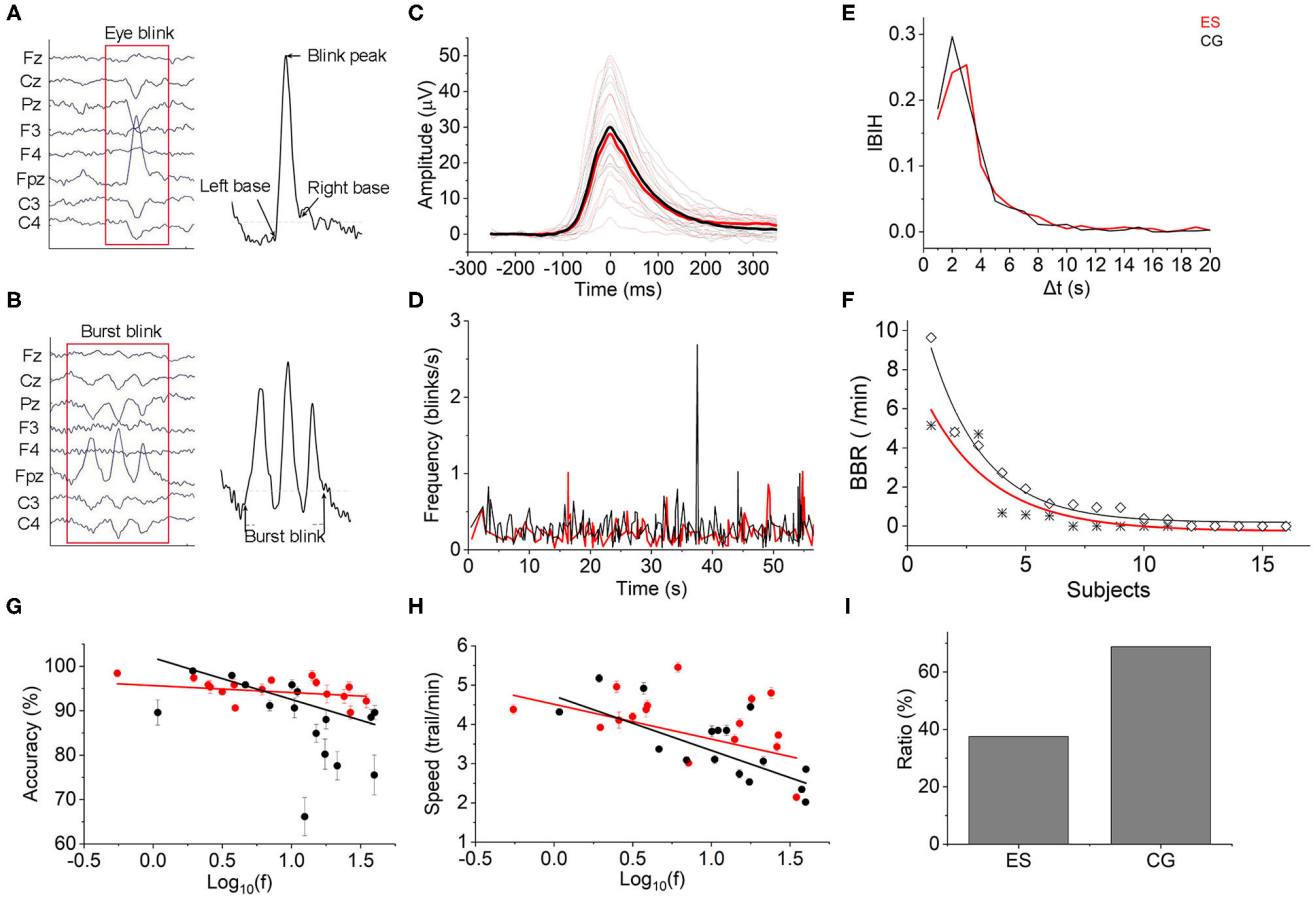
Eye blinks. **(A)** Eye blink diagram from EEG recording data. **(B)** Diagram of burst blink based on EEG data. **(C)** The mean task-evoked blink potentials of ES were lower than those of CG after Z-score normalization. Mean group potential, thick line; individual subjects' potentials, thin lines. **(D)** The mean instantaneous blink frequency of the 2 groups showed different patterns over time from the beginning to 60 ms of the CR task. **(E)** Most interblink interval duration was distributed in <20 s. The IBIH of ES was lower than that of CG, especially at the short interblink interval ( ≤ 5 s). **(F)** The BBR of ES (*) was lower than that of the controls (♢). **(G)** Blink frequency and reaction accuracy relationship. There was a negative correlation between them in both the ES and CG [ES: Accuracy= 95.662–1.566 × log10(f), *r*(16) = −0.252; CG: Accuracy = 101.892–9.356 × log10(f), *r*(16) = −0.756]. **(H)** Blink frequency and reaction speed accuracy relationship. Negative correlations were also shown in the 2 groups [ES: Speed = 4.513–0.887 × log10(f), *r*(16) = −0.492; CG: Speed = 4.723–1.386 × log10(f), *r*(16) = −0.77]. **(I)** Burst blinks appeared in 6 elite swimmers (37.5%) and 11 controls (68.75%).

The EEG-based blink amplitude and blink frequency of ES were both lower than those of CG, although the absolute values of the 2 groups were not significantly different during the CR task (Figures 6A–C). The interblink interval histogram (IBIH) also revealed that the blink frequency of ES in the short interval group was lower than that of CG (Figure 6E). From the beginning of the tasks to the following 60 s, the task-related blink rate of ES showed a regular periodic concentrated blink pattern with an interval of ~15–16 s. However, in the CG, there were continuous blinks in short periods but no obvious concentration pattern. Meanwhile, the blink frequency of CG was almost higher at each time point than that of ES during the first 1 min (Figure 6D). Interestingly, there were some burst blinks (more than or equal to 2 blinks in 1 min) in the 2 groups during the CR task. However, ES had more burst blinks than CG in both the burst blink rate (Figure 6F) and the proportion of subjects (Figure 6I).

To test whether the performance of the CR task was affected by the blinks, we computed Pearson correlation coefficients to evaluate the relationships between the complex reaction accuracy or speed and blink frequency. As shown in Figure 6G, there was a negative correlation between log_10_ blink frequency and complex reaction accuracy in both groups. However, a smaller slope was displayed in the ES than in the CG, which indicated that the task performance of master swimmers was less influenced by the blinks. Consistent with the reaction accuracy, the complex reaction speed also exhibited a negative linear relationship with log blink frequency but was less affected than the reaction accuracy in terms of the slope difference (Figure 6H).

## Discussion

Our findings showed that elite swimmers were significantly better in performing the eye-hand-foot reaction task while showed less energy-cost wiring connections than age-matched college students. Elite athletes also had highly stabilized eye blinking rate. These results suggest that long-term professional training of arm-leg coordination may facilitate formation of necessary direct wire connections whose number is significantly less than controls. This may be an energy saving for the brain and would enhance the reaction time and keep the brain focus on the task in hand.

Due to long-term professional skill training, athletes are expected to have some phenotypic characteristics (Simonek, 2011). Here, our study showed that elite swimmers had significantly faster reaction speed and more accurate responses to complex reactions than age-matched college students with no professional swimming training. It was consistent with many previous studies (Mori et al., 2002; Williams et al., 2002; Kida et al., 2005). As the number of choices increases, the probability of differences between individuals also increases (Hick, 1952). Complex reaction tasks require not only attention and exercise execution but also the ability to discriminate stimulus's features and to make response selection in a fast way (Miller and Low, 2001). The performance is determined by many factors, such as age, gender, physical activity, and training (Spirduso, 1975; Morehouse and Miller, 1976; Davranche et al., 2006; Enel and Erog, 2006). A 6-week training program could significantly reduce the reaction time of the peroneal muscles of healthy subjects, which might be related to the improvement of reaction inhibition ability by exercise training (Linford et al., 2006). When a selection error occurred in the complex reaction, the individual would spend more time in response suppression to limit the recurrence of the error (Welford, 1988; Koehn et al., 2008). Due to the long-term cooperative training of hands and feet for underwater resistance, the inhibition and control capabilities of swimmers might be improved. Therefore, complex reaction performance is regarded as a behavior characteristic for the selection and cultivation of elite swimmers (Guang et al., 2013).

In addition, a highly efficient mammalian brain is generally shaped with optimal wiring connections of cortical units to support high-profile cognitive functions (Laughlin and Sejnowski, 2003). Such economical wiring networks are developed from long-term learning and shaped by energetically costly spatially distributed spiking and synaptic activities (Alle et al., 2009; Carter and Bean, 2009; Yu et al., 2012) that could be captured by EEG recordings on the scalp. We observed that with almost equal EEG activity levels, the brains of elite swimmers exhibited more decorrelated network connectivity than the control group in the frontoparietal region at the specific upper β frequency band (20–30 Hz). Interestingly, within ES group, the more wiring connections, the longer training years which positively correlated with CR performance. It indicated that the least wiring connections didn't mean the best performance. There was a balance between them and existing the preferred wiring number of connections during the given task. Previous study has shown that wiring number of connections is highly correlated with the energy cost (Tomasi et al., 2013). The frontoparietal network is observed to be a “fingerprint” that can predict cognitive efficiency or intelligence level (Finn et al., 2015). Thus, functional connectivity with lower intensity and variance in athlete frontoparietal brain may suggest the underlying energy-efficient wirings.

In the other low-frequency EEG bands, the wiring connections and activity cost of elite swimmers were not significantly different from those of the control group. These results indicated that the β frequency band played a key role for the master swimmers. Previous studies have found that the β frequency band is important in sensorimotor integration (Vukelic et al., 2014), attention processing (Chung et al., 2017), or sensory functions such as somatosensory input (Pfurtscheller et al., 2001). The suppression of the β frequency band was significantly related to the reduction in reaction time (Pollok et al., 2014) and response error (Chung et al., 2017). In our research, however, we found that only when the β frequency band was subdivided into lower (13–20 Hz) and higher (20–30 Hz) β frequency bands did the functional connectivity of elite swimmers appear to be significantly different from that of the control group. This result suggested that top swimming athletes might apply different frequency selective strategies when performing complex reaction task.

In addition, in the β frequency band, top swimming athletes were more inclined to focus on the upper β frequency band with greater power changes and less correlated functional connectivity. In the control group, both the lower and upper β frequency bands had wide-ranging power changes with relatively small amplitudes and strong functional connectivity. The pattern of increasing the power changes of the high-frequency band and reducing the corresponding functional connectivity might be one of the reasons why elite swimmers exhibited higher network wiring cost-efficiency. Moreover, the UBTR changes of the elite swimmers were significantly different from those of the control group in the frontal and central parietal areas. It was mainly manifested in the interactive phenomenon that the UBTR of elite swimmers increased while that of the control group decreased in the left and right frontal and central parietal areas. This further illustrated that the spectrum power changes were different between the elite swimmers and the control group in the low- and high-frequency bands. From the resting state to the task state, top swimming athletes tended to increase spectral power and less correlated functional connectivity to reduce network wiring costs and optimize wiring cost-efficiency.

In the present study, we also found that the parietal lateralization index of the elite swimmers was significantly higher than that of the control group and was closer to 0 during the CR task. The lateralization index reflected the power changes of the left and right hemispheres in the α frequency band (Neubauer et al., 2020). The lower the power of the α frequency band was, the more highly the brain was activated (Bazanova and Vernon, 2014). Therefore, elite swimmers seemed to show more balanced brain activation and higher activation of the right hemisphere. It was demonstrated that leftwards asymmetries were present in the motor control regions and that motor response areas, such as the precentral gyrus, supplementary motor area and several basal ganglia, were initiated in right-handed subjects (Rogers et al., 2004; Dadda et al., 2006; Luders et al., 2006; Toxopeus et al., 2007; Coxon et al., 2010). Our results suggest that the brain activities in the elite swimmers were more bilateral during the complex reaction task, indicating an increased symmetry of 2 hemispheres due to the long-term and regular coordinated movement of arms and legs.

Blink rate was proposed to serve as a non-invasive, indirect measure of dopamine (DA) activity in the central nervous system and has been used to study cognitive control, learning, working memory, and decision making (Eckstein et al., 2017). A previous study observed that higher blink rates predicted better performance on set-shifting and Stroop tasks but worse performance on an updating task (Zhang et al., 2015). Other research revealed that a higher blink rate was related to lower distractibility on tasks that place high demands on working memory (Colzato et al., 2009). In our study, a lower task-related blink rate in elite swimmers showed better performance on complex reaction task, which was different from the Stroop tasks but consistent with the research that blinks affect the performance of visual attention (Cruz et al., 2011). Moreover, the CR task involves many brain regions for vision, motion, planning, cognitive computing, attention, and decision making and may also be closely correlated with DA functions (Westbrook and Braver, 2016). An inverted U-shaped relationship between DA and cognitive control (Goldman-Rakic et al., 2000) indicated that the best cognitive control ability was related to preferred DA activity. That is, a blink rate that is too high might result in worse cognitive control. However, to what extent the blink rate was the best is still open. In light of varying testing- and participant-related affective factors (Eckstein et al., 2017), it is suggested that methods and conditions should be cautiously selected when using blink amplitude or frequency as a biomarker. Moreover, Ponder and Kennedy (1928) noticed that the interblink intervals were quite variable between subjects. Due to the short recording time in our study, there were no significantly different interblink interval distribution patterns between the 2 groups (Supplementary Figure 9). Whether the pattern of interblink interval distribution is a biomarker for elite athletes needs to be further cautiously designed and long-term recorded research.

The main limitations of this study were as follows: (1) There were only 8 frontoparietal channels for recording EEG signals, so the brain activities and cost-efficiency of other brain regions are not accessed; (2) the brain cost-efficiency was based on the strength of functional connectivity and mean frequency rather than direct measurement based on blood oxygen or glucose metabolism, which might lack quantitative accuracy. (3) Master-level swimmers were very rare and distributed to 5 main items, so we cannot classify the events in the light of stroke and compared the competition results within the swimmer athlete group.

## Conclusion

In summary, elite swimmers are faster and more accurate in performing the complex reaction task than controls. By using wiring number of connections between EEG channels to represent wiring cost and mean frequency to index the activity cost of brain, we found that elite swimmer's brain has less and weaker correlations among frontal and parietal regions in upper beta frequency band than controls. This finding suggests that elite swimmers' brains are more energy efficient in wiring connections. However, the mean activity rates of elite swimmers' brains are slightly higher than controls' (although the difference is not significantly), suggesting that they are more actively in response to the task. Meanwhile, athletes showed higher stability and lower eye-blinking rate comparing to controls. A set of distinct physiological features, e.g., energy-efficient wiring connections and stable blinking dynamics, together with behavior performance results, could potentially be used as effective measures, to identify athletes with great potential in achieving competitive performance.

## Data Availability Statement

The datasets presented in this study can be found in online repositories. The names of the repository/repositories and accession number(s) can be found at: https://osf.io/4mzra/.

## Ethics Statement

The studies involving human participants were reviewed and approved by the Ethics Committee of the Fudan University. The patients/participants provided their written informed consent to participate in this study.

## Author Contributions

YY and XP conceptualized and designed the study. XP, XQ, and YC collected data. XP and YJ analyzed data and interpreted results. XP wrote the paper. A-LW commented on the paper. CZ, YY, and XS supervised the research. All authors reviewed and approved the final version of the manuscript.

## Conflict of Interest

The authors declare that this study received resources from Shanghai PsyTech Electronic Technology Co. Ltd. The company was not involved in the study design, collection, analysis, interpretation of data, the writing of this article or the decision to submit it for publication.
