# Supplementary material for: Sparsely Wiring Connectivity in the Upper Beta Band Characterizes the Brains of Top Swimming Athletes
Source: Front Psychol. 2021 Jul 16;12:661632. doi: 10.3389/fpsyg.2021.661632 (PMC8322235; doi:10.3389/fpsyg.2021.661632)
Supplement: Supplementary file 1 [file Data_Sheet_1.pdf]

Supplementary Information

Supplementary Methods

Method S1. Alpha Blocking Rate

The alpha rhythm is the prominent EEG wave pattern of an adult who is awake but relaxed with eyes closed. The amplitudes of alpha waves diminish when subjects open their eyes. This is called the alpha-blocking effect(Zheng et al., 2018). The alpha-blocking phenomenon is conceptualized as desynchronized neural population activity during active stimuli, and alpha blocking rate ($\alpha_{\text{blocking rate}}$) between the EC and EO states is used to monitor the stability of the experimental recording process. We calculated $\alpha_{\text{blocking rate}}$ as follows:

$$\alpha_{blocking\,rate} = \frac{\left(log_{10}(P_{EC}(f)) - log_{10}\left(P_{EC\_base}(f)\right)\right) - (log_{10}(P_{EO}(f)) - log_{10}\left(P_{EO_{base}}(f)\right))}{(log_{10}(P_{EC}(f)) - log_{10}\left(P_{RC_{base}}(f)\right))} \times 100\% \quad (1)$$

Where P_{EC}(f) and P_{EO}(f) represent power spectrum of recorded EEG signals. P_{EC_base}(f) and P_{EO_base}(f) was the baseline defined the mean power in the frequency range of 7-13Hz for each channel and then averaged over 8 channels.

Method S2. Lateralization Index

The human brain has different regions to handle specific tasks.(LeMay, 1977) This lateralization is widely considered a key characteristic of the brain that contributes to higher cognitive functions.(Neubauer et al., 2020) In the language region there is typical left-brain lateralization,(Tzourio-Mazoyer et al., 2004) whereas right lateralization is in the attention control network.(Nielsen et al., 2013) Frontal or parietal asymmetry of EEG alpha power may index the risk for anxiety or depression under large pressure, especially for young adults.(Smit et al., 2007) The most commonly reported measure for lateralization index (LI) is computed by subtracting the alpha power of right hemisphere from the alpha power of left hemisphere alpha power:

$$\mathrm{LI} = \frac{P_{left\_alpha} - P_{right\_alpha}}{P_{left\_alpha} + P_{right\_alpha}} \quad (2)$$

Where *P* means power. In interpreting this scale, lower scores indicate relatively greater left frontal or parietal activation whereas higher scores indicate relatively greater right frontal or parietal activation (please note here high alpha power indicates the putative inverse of activation of brain activities). A bunch of studies observed that greater relative left frontal activation might be expected to be associated with greater self-reported happiness or less anxiety.(Harmon-Jones and Allen, 1997; 1998) Activation of the left parietal region and bilateral in the premotor cortex were reported during planning and executing praxis movements focusing on tool-use pantomime.(Maki-Marttunen et al., 2014) Abundant evidences identified a relative lateralization toward the right hemisphere of some of the cortical networks supporting the attention systems, especially those including the temporo-parietal junction and the ventrolateral prefrontal cortex.(Bartolomeo and Seidel Malkinson, 2019) Here PF3 or PC3 was taken as $P_{left\text{-}alpha}$ and PF4 or PC4 was taken as $P_{right\text{-}alpha}$ for calculating frontal lateralization

Index (LI_f) or parietal lateralization Index (LI_p).

Method S3. Blink Recognition Procedure and Pseudo Code

Three other conditions were taken into consideration during the following procedure, that is, 1) the heights of the peak should be larger than a threshold in the Fpz channel; 2) there should be no less than three channels having throughs; 3) if the inter peak interval between two peaks are smaller than a threshold and the minimum amplitude in this interval is not small enough, the smaller peak should be removed. No other conditions were added when using the function *findpeak* in Matlab. In order to better explain this process, we added the following pseudo code:

```
procedure BlinkRecognition
potential_peaks ← find potential peaks in the Fpz channel using the function findpeak in Matlab
for peak in potential_peaks do
    heights[peak] ← compute the height of this peak
end for
height_max ← max(heights)
for peak in potential_peaks do
    if heights[peak] < 2 / 5 * height_max do
        remove this peak
    end if
    if there are no more than 2 channels having through at the same period do
        remove this peak
    end if
end for
n ← the number of peaks
for i = 2, 3, 4, …, n do
    if the time interval between peaki-1, peaki < 500 ms do
        mid ← the minimum amplitude in the Fpz channel in this time interval
        if amplitude(peaki-1) – mid < 1 / 3 * height_max or amplitude(peaki) – mid < 1 / 3 *
height_max do
            remove the smaller peak
        end if
    end if
end for
the remaining peaks are regarded as the peaks related to eye blinks
return peaks
```

Method S4. The procedure and code of muscle artifact detection and removal

Firstly, each subject was selected 50 seconds signals of EC state, 50 seconds signals of EO state, and 70 seconds signals of CR task (selection from the beginning recording signals of the CR task) to be preprocessed. The mean frequency of 25 segments or 35 segments EEG data from 8 channels were calculated in 2 seconds.

Secondly, we calculated the Ratio of each segment from each channel of every subject using MATLAB script according to the formula (1) in the manuscript.

Thirdly, when the value of Ratio of one segment from one channel of a subject was greater than 1, this segment was removed and the mean frequency of this channel of one subject would be calculated according to the left segments.

Finally, the mean frequency of each channel of ES or CG would be calculated in the condition of EC, EO or CR respectively. Eighteen segments of EC were removed from 800 segments (25*32=800, 2.25%), fifty segments of EO were removed from 800 segments of 2groups (25*32=800, 6.88%) and sixty-two segments of CR were removed from 1120 segments of 2 groups (70*32=1120, 5.54%).

MATLAB code script:

```
function [MF,Ratio,time_index]=averf2s_highlowratio(x,F0)
mn=size(x);
for i=1:mn(1)
    x(i,:)=detrend(x(i,:));
end

nfft = 1024;
for i=1:mn(1)
      for j=1:floor(mn(2)/2000)
       d=x(i,(j-1)*2000+1:j*2000);
       [px(j,:),fxi] = pwelch(d,hanning(nfft),nfft/2,nfft,F0);
       nx1=find(fxi>0 & fxi<=13);P1=sum(px(j,nx1));
       nx2=find(fxi>13 & fxi<=30);P2=sum(px(j,nx2));
       Ratio(i,j)=P2/P1;
       If (Ratio(i,j)<1)
           time_index(i,j)=1;
       else
           time_index(i,j)=2;
       end
       mf(j) = sum(px(j,:).*fxi')./sum(px(j,:));
       end
       MF(i,:)=mf;
end
end
```

This method of muscle artifact removal was actually from our lab but it was based on some characters of muscle artifact and some similar methods.

First, the two references we have indicated support that EMG artifacts have a higher amplitude than the EEG signals.

Second, in a muscle component part of ICLabel Tutorial, it is summarized that power of muscle component concentrated in higher frequencies. (https://labeling.ucsd.edu/tutorial/labels) In a previous study, there was a figure also supporting this character of muscle artifact. (Goncharova et al, 2003).

Third, in a study about automatic artifact removal (AAR) toolbox for MATLAB (Gomez-Herrero, 2007), they use an important parameter of the *emg_psd* criterion that is the ratio of average power in the typical EEG band and in the typical EMG band. (page 11).

Here, we show an example session in Figure S14 with our method of muscle artifacts removal. In Figure S15A, we can see the muscle artifacts exist mainly in the fourth segment (6-8 s). When using our method, there were 2 ratios, in C3 and C4 channels, greater than 1 in the fourth segment (Figure S15B).

Meanwhile, we processed the CR task EEG data of 32 participants of 2 groups. There were altogether 8960 ratios of all segments (35*8*32=8960). Most segments of EEG data (8897 segments) didn't show obvious muscle artifacts after filtered. Sixty-three ratios were greater than 1, 74.6% of which were distributed from 1 to 2. About 95% of brain signals were distributed from 0 to 0.5. The log10 counting distribution and normalized histogram of the ratios were shown in Figure S15C. It was reasonable for being set 1 as the ratio threshold to detect the muscle artifacts.

Therefore, our method of muscle artifacts removal can be used to detect muscle artifacts especially when the channels were not enough to run ICA.

Supplementary Figures

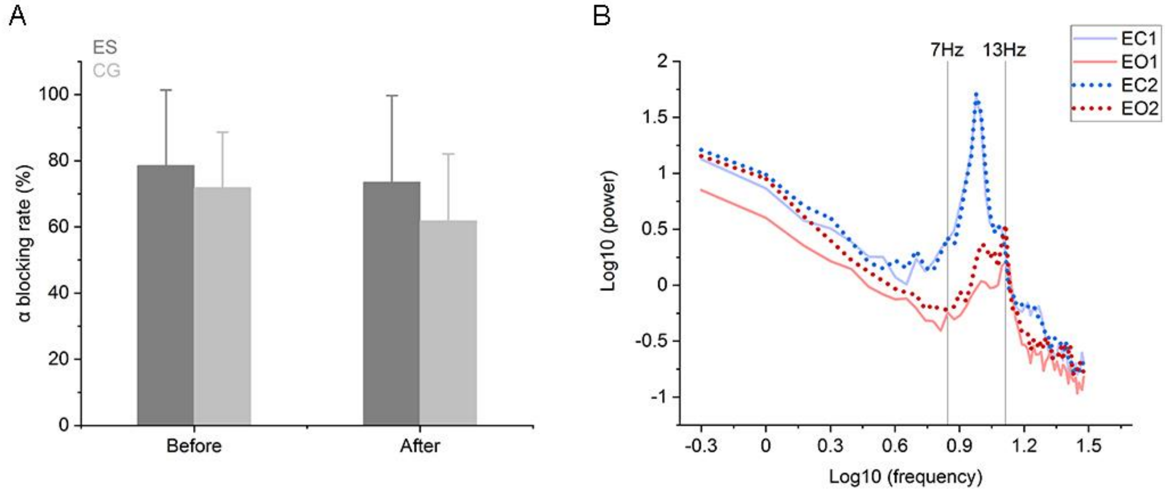

Figure S1. Schematic diagram of $\alpha_{blocking\ rate}$. (**A**) After the CRtasks, the $\alpha_{blocking\ rate}$ of the 2 groups, ES and CG both had a downward trend due to the impact of the task, but the decline rates were less than 20%. (**B**) This was 1 subject's schematic diagram of $\alpha_{blocking\ rate}$ before and after the CR- task.

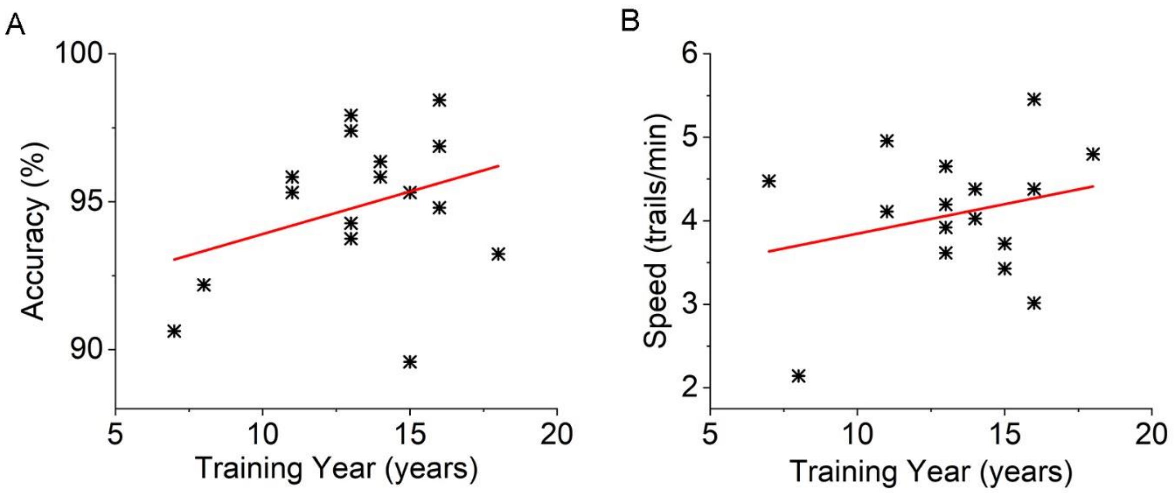

Figure S2. The relationship between CR performance and swimming training year. (**A**) CR accuracy of ES was positively correlated with their swimming training year (T). ($Accuracy_{ES} = 91.033 + 0.287T$, r (16) = 0.336). (**B**) There was a positive linear relation between the CR speed and training year in the ES group. ($Accuracy_{CG} = 3.139 + 0.07T$, r（16）= 0.26).

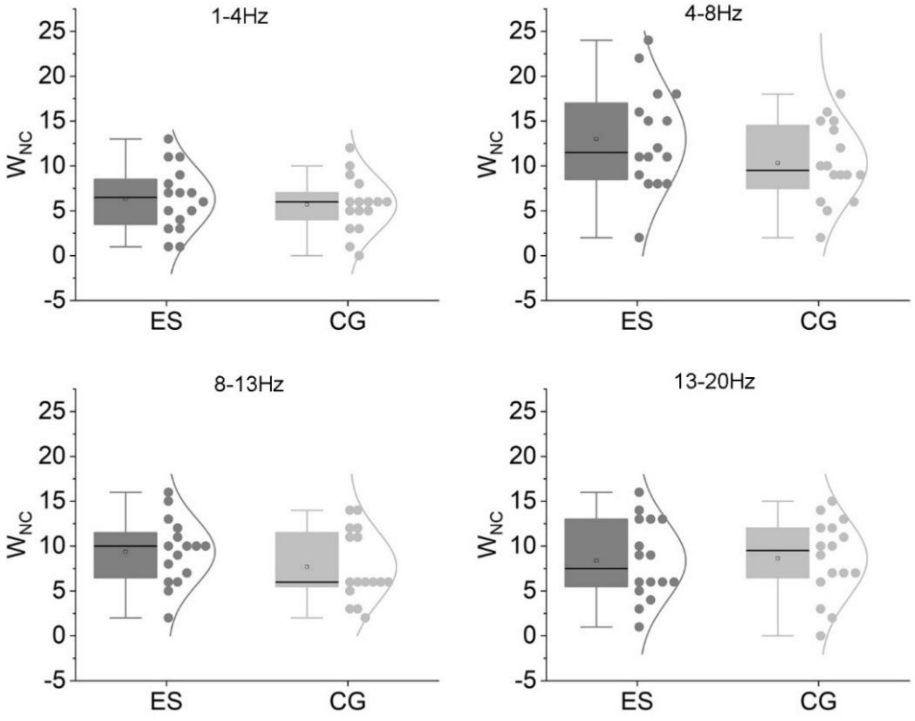

Figure S3. W_{NC} of 2 groups showed no significant differences at the δ, θ, α and lower β frequency bands during the CR task (p>0.05). Each dot represents the W_{NC} data of one subject.

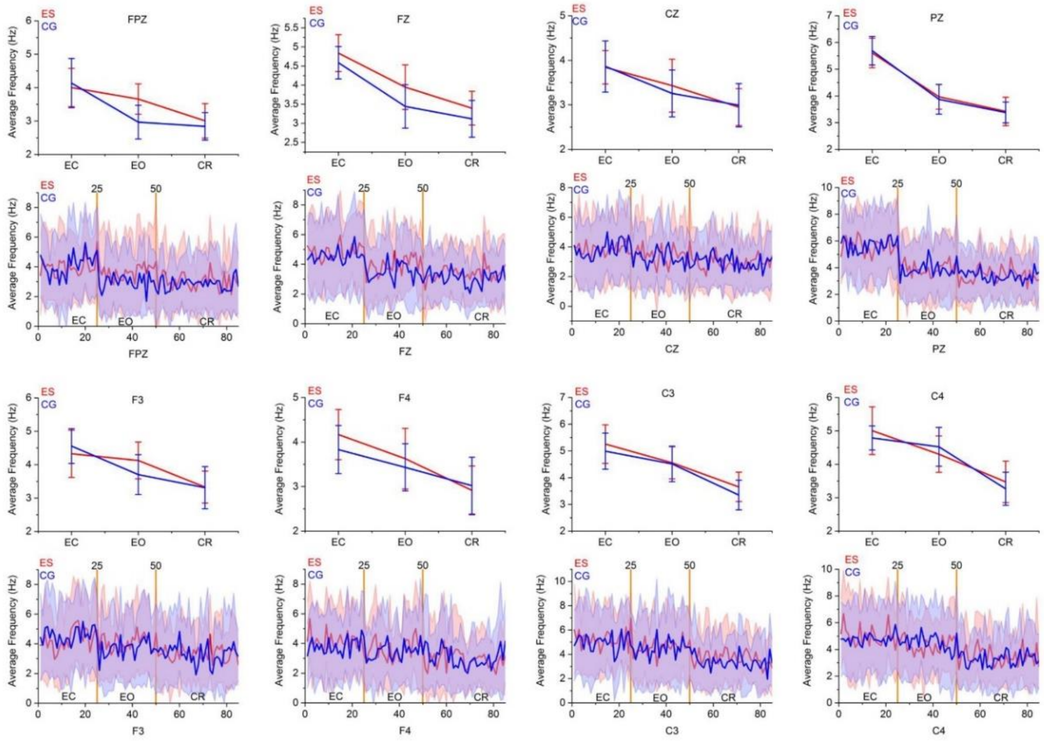

Figure S4. The trends of mean frequency at 8 frontoparietal channels from the EC state to the EO state then to the CR task state.

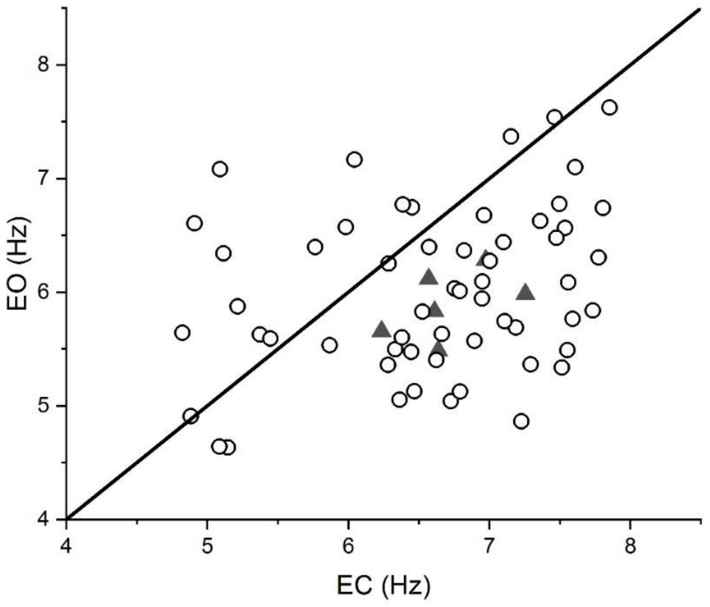

Figure S5. The mean frequency of 64 channels of 9 healthy persons in our lab's previous study under the resting EC state and the EO state. The 6 electrodes shown by the triangle symbols, were same like the electrodes selected in this experiment, exhibited a decrease trend from the EC state to the EO state, which was consistent with our current research result.

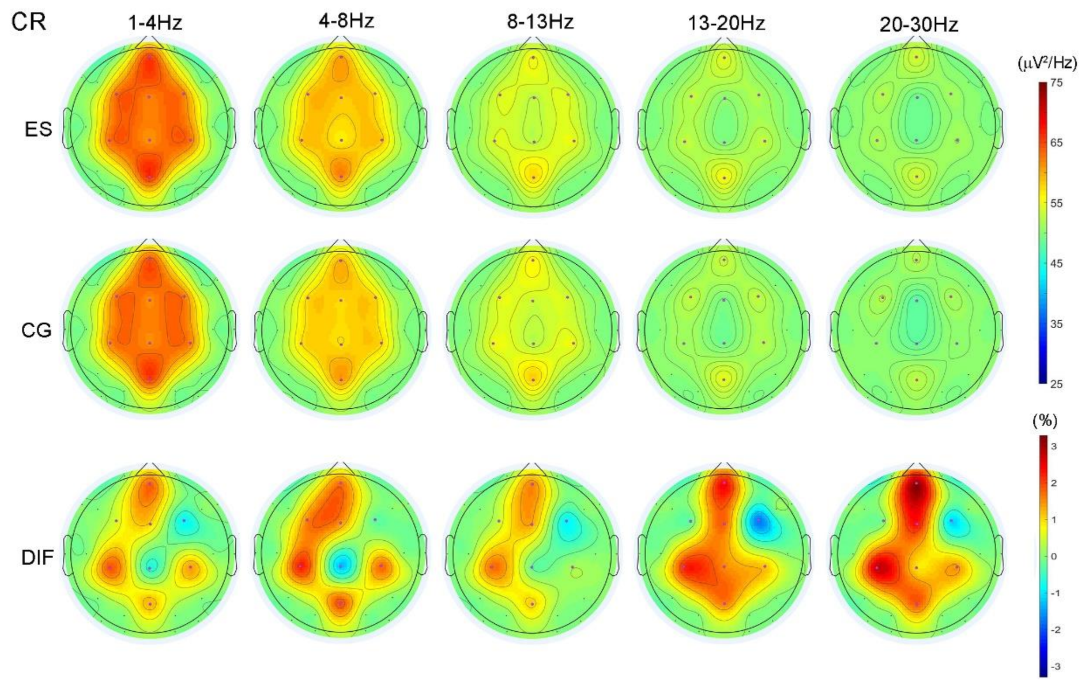

Figure S6. Spectrum power topologies of the 2 groups at 5 different frequency bands during the CR task.

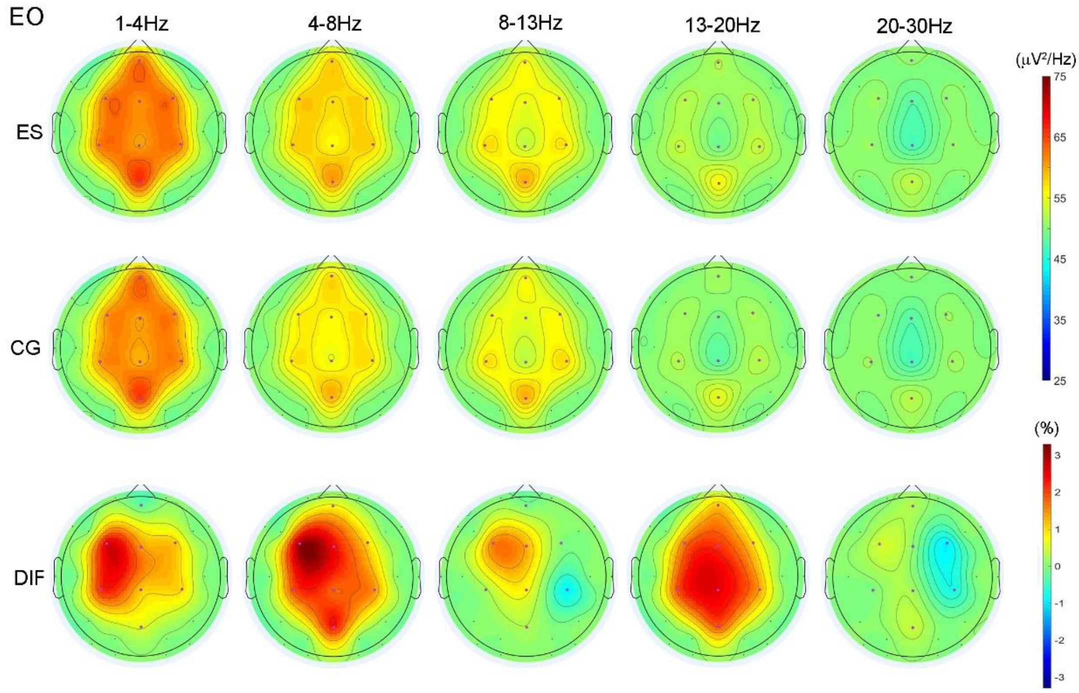

Figure S7. Spectrum power topologies of the 2 groups at 5 different frequency bands in the resting EO state.

(Note that in the Supplementary Fig. 6 and Supplementary Fig. 7, only the 8 purple-labeled electrodes in the frontoparietal region were tested by our experiment, while other 18 black-labeled electrodes in the surrounding green area were added by us in the drawing, whose power value were all set to 0, in order to avoid the influence of the frontoparietal signals on the periphery.)

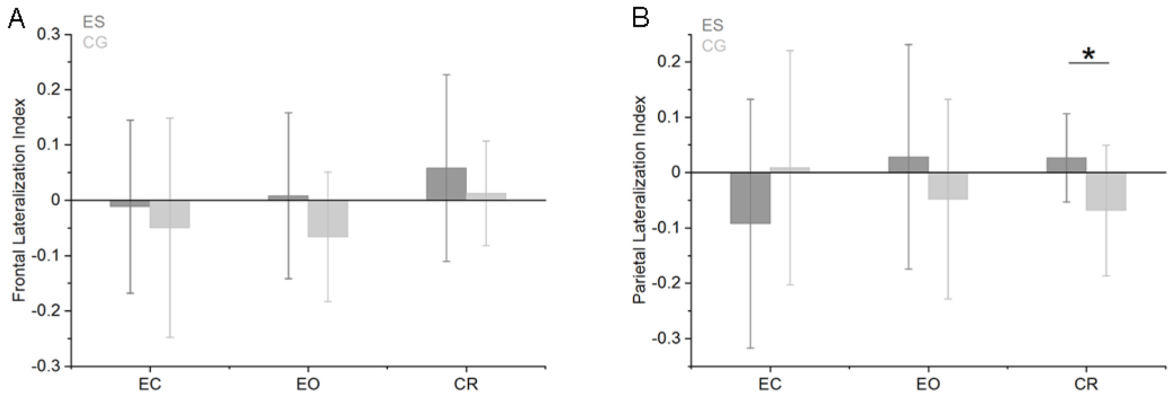

Figure S8. The lateralization index of the 2 groups in the resting and task states. (**A**) There was no significantly statistical difference in the frontal lateralization index between the ES and the CG neither in the EC state nor in the EO state or in the CR task state. (**B**) In the CR task state, the parietal lateralization index of the elite swimmers was significantly different from that of the control group. However, in the other 2 resting states there was no significantly statistical difference between the 2 groups, * $p<0.05$.

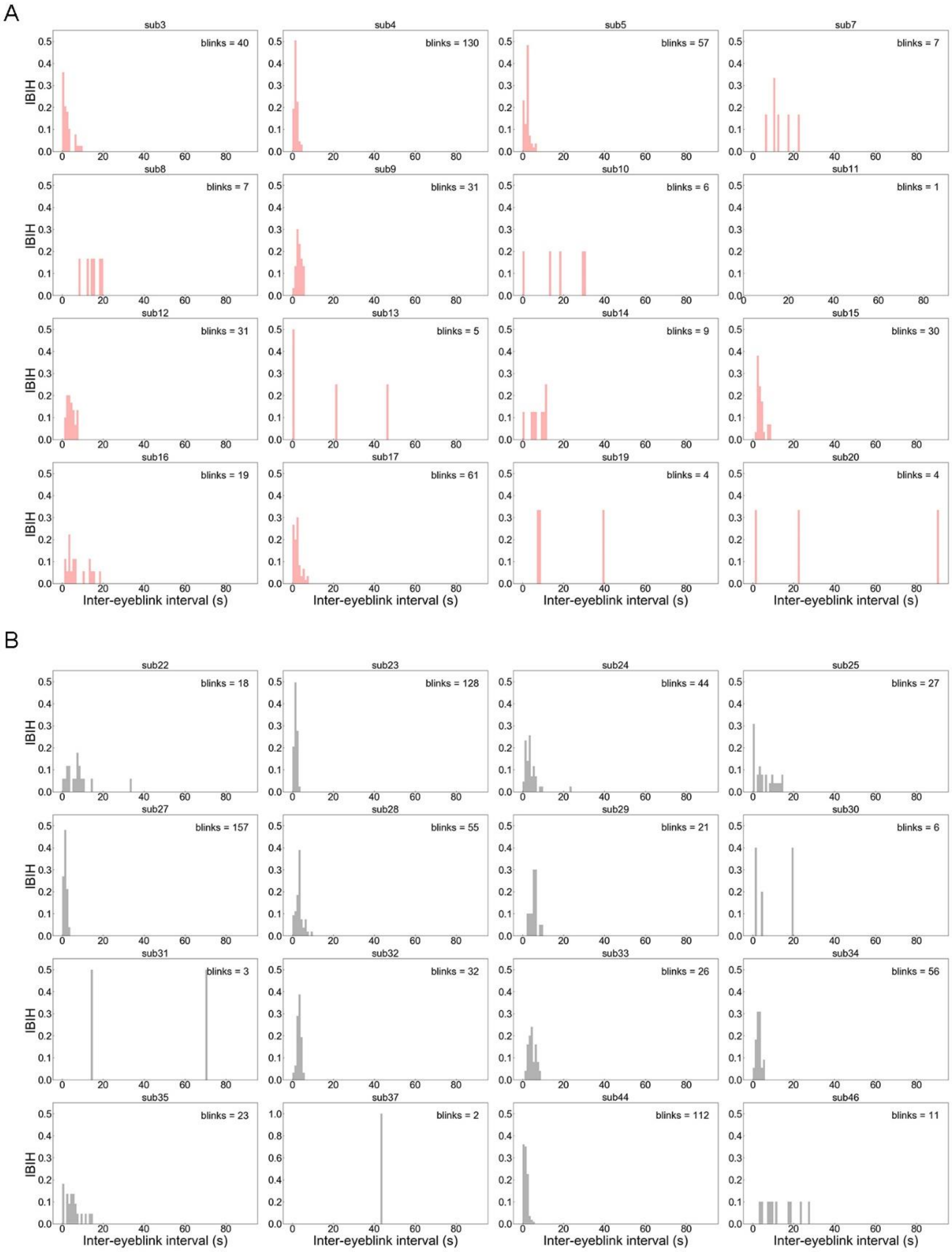

Figure S9. Interblink interval (IBI) distributions. (**A**) IBI distributions of 16 elite swimmers during the complex reaction task. (B) IBI distributions of 16 age-matched college students during the complex reaction task.

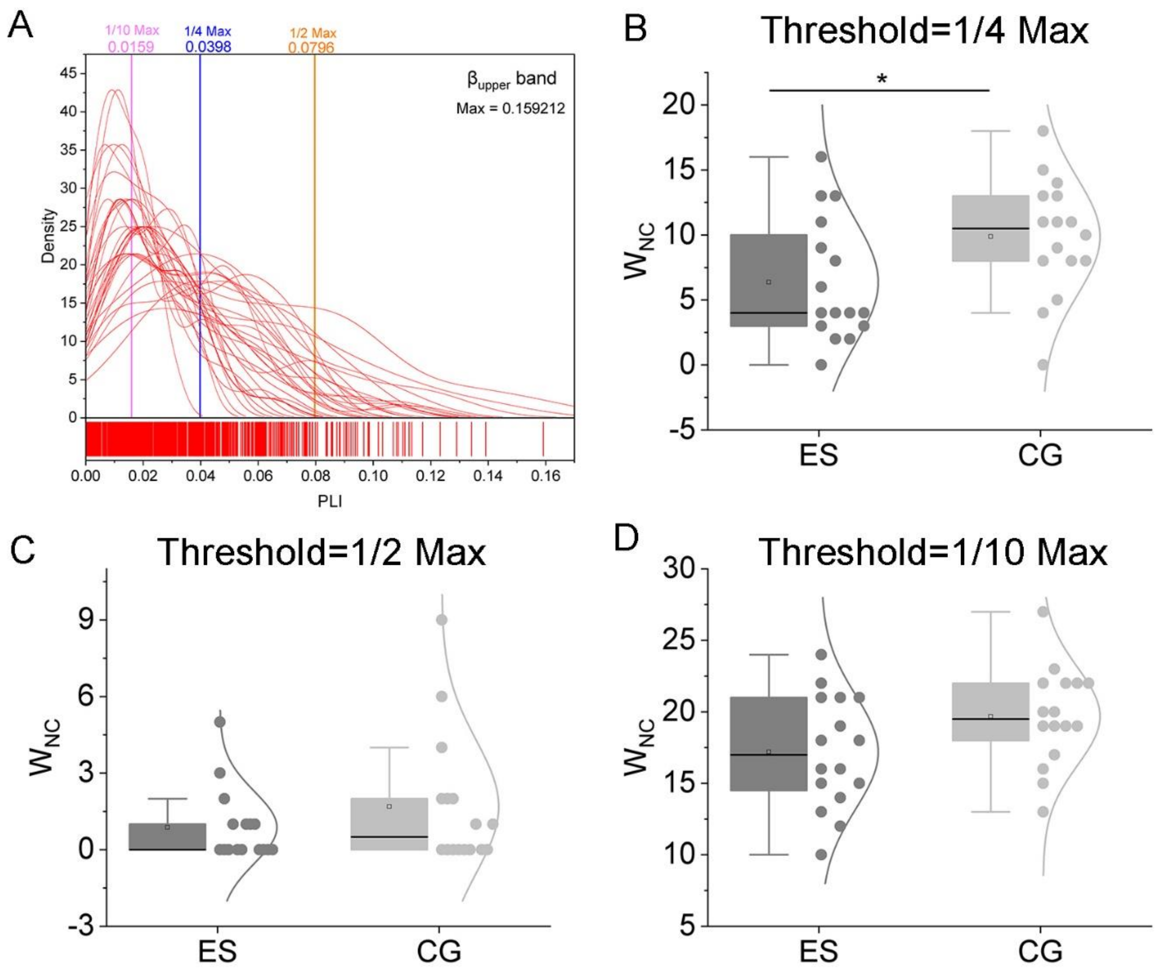

Figure S10. **The comparison of different threshold in the upper beta frequency band.** (**A**) PLI value of each subject distribution. The maximum PLI value of all participants (Max) is calculated. Blue line represents 1/4 Max, pink and orange line means 1/10 Max and 1/2 Max respectively. (**B**) There was significantly different between ES an CG when the threshold of PLI value was set as 1/4 Max. (**C**) No significantly different between two groups when the threshold of PLI value was set as 1/2 Max. (**D**) There was no significant differences between two groups when the threshold of PLI was set as 1/10 Max.

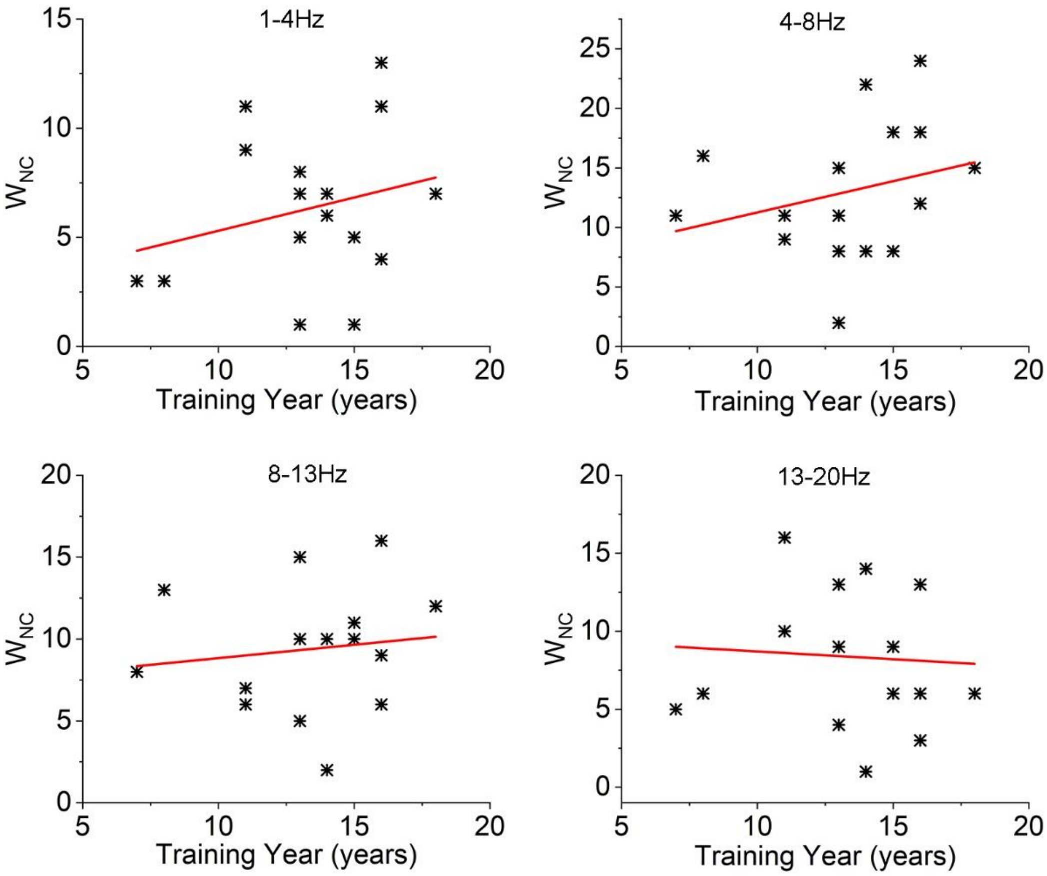

Figure S11. The correlation between W_{NC} and swimming training years (T) in the other four frequency bands. ($W_{NC1\text{-}4Hz} = 2.256 + 0.305$ T，$r(16)_{1\text{-}4Hz} = 0.253$；$W_{NC4\text{-}8Hz} = 6.006 + 0.525$ T, $r(16)_{4\text{-}8Hz} = 0.269$; $W_{NC8\text{-}13Hz} = 7.202 + 0.163$ T, $r(16)_{8\text{-}13Hz} = 0.13$; $W_{NC13\text{-}20Hz} = 9.699 – 0.099$T, $r(16)_{13\text{-}20Hz} = -0.066$)

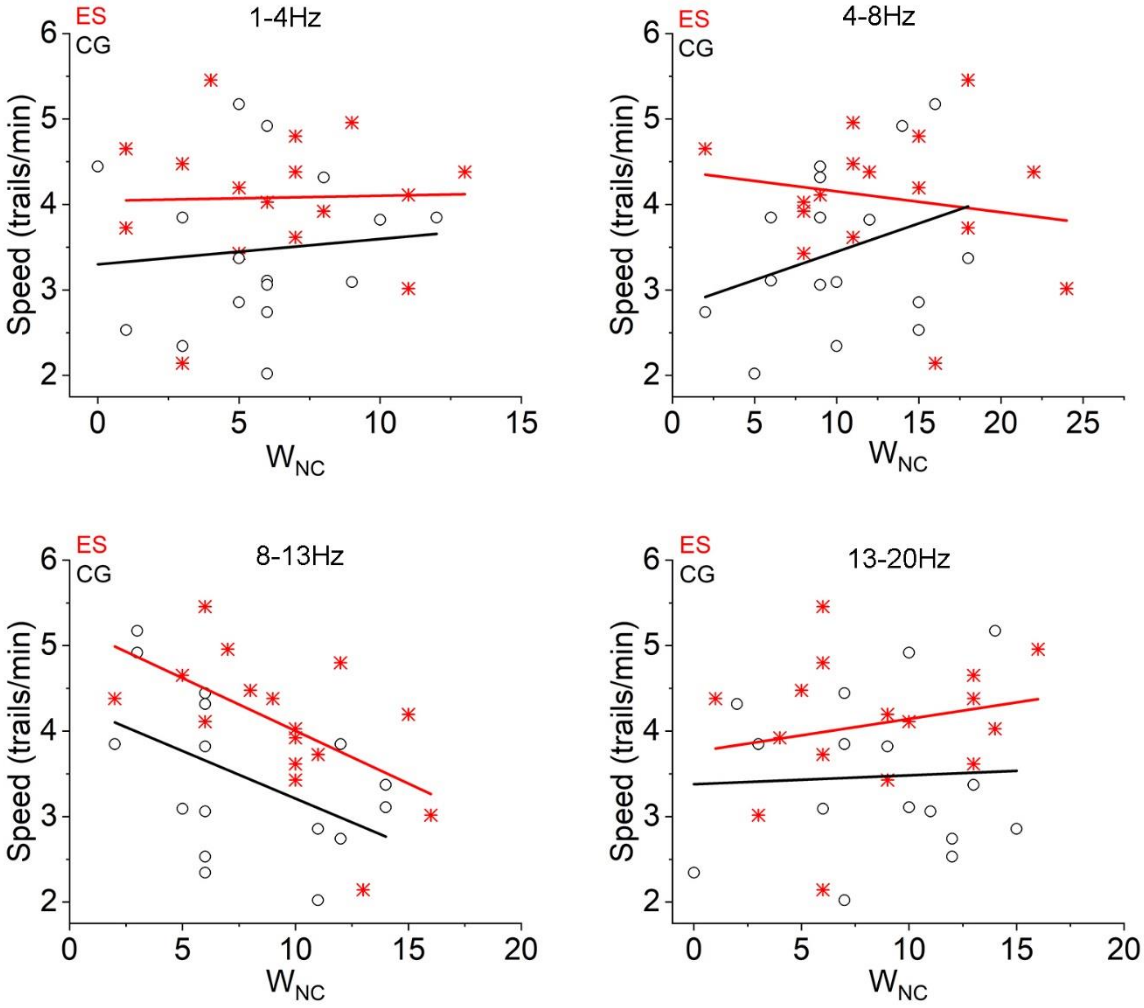

Figure S12. The correlation between W_{NC} and the CR speed (S) in the ES and CG groups at the other four frequency bands. ($S_{ES1\text{-}4Hz}$ = 4.043 + 0.006 W_{NC}, $r(16)_{ES\_1\text{-}4Hz}$ = 0.026; $S_{CG1\text{-}4Hz}$ = 3.299 + 0.03 W_{NC}, $r(16)_{CG\_1\text{-}4Hz}$ = 0.101; $S_{ES4\text{-}8Hz}$ = 4.398 – 0.024 W_{NC}, $r(16)_{ES\_4\text{-}8Hz}$ = -0.176; $S_{CG4\text{-}8Hz}$ = 2.786 + 0.066 W_{NC}, $r(16)_{CG\_4\text{-}8Hz}$ = 0.318; $S_{ES8\text{-}13Hz}$ = 5.237 – 0.123 W_{NC}, $r(16)_{ES\_8\text{-}13Hz}$ = -0.57; $S_{CG8\text{-}13Hz}$ = 4.324 – 0.111 W_{NC}, $r(16)_{CG\_8\text{-}13Hz}$ = -0.484; $S_{ES13\text{-}20Hz}$ = 3.757 + 0.039 W_{NC}, $r(16)_{ES\_13\text{-}20Hz}$ = 0.214; $S_{CG13\text{-}20Hz}$ = 3.378 + 0.01 W_{NC}, $r(16)_{CG\_13\text{-}20Hz}$ = 0.049.)

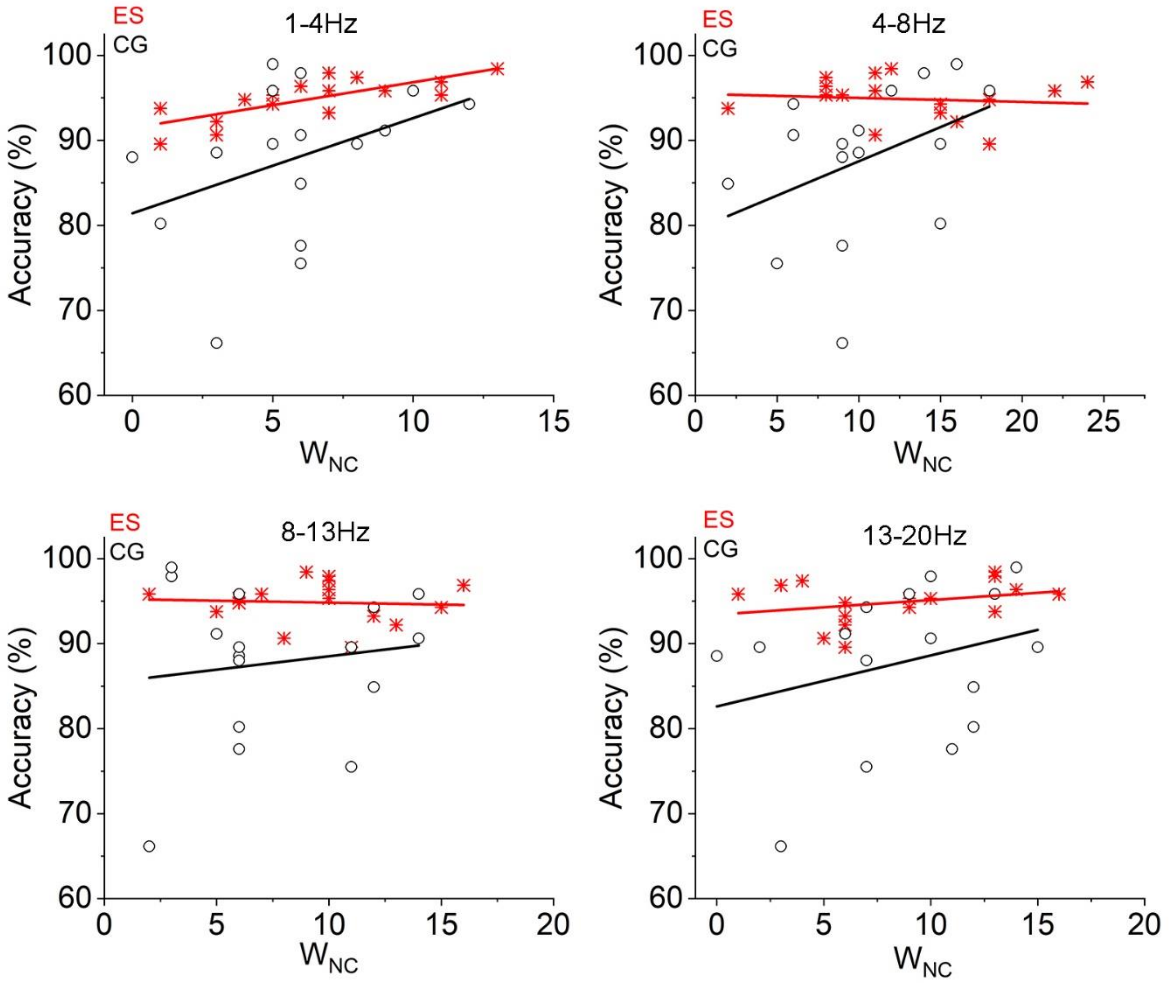

Figure S13. The correlation between W_{NC} and the CR accuracy (A) in the ES and CG groups at the other four frequency bands. ($A_{ES1\text{-}4Hz} = 91.465 + 0.537\ W_{NC}$, $r(16)_{ES\_1\text{-}4Hz} = 0.757$; $A_{CG1\text{-}4Hz} = 81.422 + 1.12\ W_{NC}$, $r(16)_{CG\_1\text{-}4Hz} = 0.388$; $A_{ES4\text{-}8Hz} = 95.474 – 0.047\ W_{NC}$, $r(16)_{ES\_4\text{-}8Hz} = -0.109$; $A_{CG4\text{-}8Hz} = 79.498 +0.804\ W_{NC}$, $r(16)_{CG\_4\text{-}8Hz} = 0.396$; $A_{ES8\text{-}13Hz} = 95.282 – 0.045\ W_{NC}$, $r(16)_{ES\_8\text{-}13Hz} = -0.067$; $A_{CG8\text{-}13Hz} = 85.371 + 0.315\ W_{NC}$, $r(16)_{CG\_8\text{-}13Hz} = 0.14$; $A_{ES13\text{-}20Hz} = 93.428 + 0.171\ W_{NC}$, $r(16)_{ES\_13\text{-}20Hz} = 0.301$; $A_{CG13\text{-}20Hz} = 82.615 +0.6\ W_{NC}$, $r(16)_{CG\_13\text{-}20Hz} = 0.291$.)

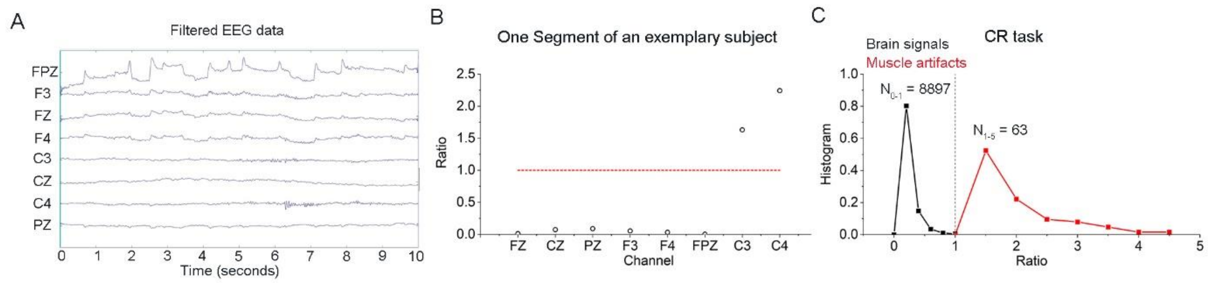

Figure S14. The method using the value of ratio to detect the muscle artifact segments during the CR task. (A) The example session (10 seconds) of filtered EEG data with muscle artifacts, which were most in the fourth segments (2 seconds per segment). **(B)** The ratios of two channels (C3 and C4), calculated by the formula (1) in the manuscript, were greater than 1 in the fourth segment. **(C)** There was the normalized histogram of 8897 segments of brain signals and 63 segments of muscle artifacts.

Supplementary Tables

Table S1. Description of participants

Participants	Test	Effective Statistics				
		All	Male	Female	Age (year)	Level
ES	21	16	9	7	19.11±2.1	Master = 13<br>National First Class = 3
CG	23	16	8	8	19.29±0.85	None
Total	44	32	17	15		

Abbreviation: CG = control group; ES = elite swimmers.

Table S2. Demographic of the elite swimmers for EEG analysis

Subject	Sex	Main Item	Training Year (year)<br>(up to the year of EEG recording)
Sub01	M	Backstroke	18
Sub02	F	Free Style	8
Sub03	M	Free Style	15
Sub04	M	Backstroke	7
Sub05	F	Breaststroke	14
Sub06	M	Free Style	13
Sub07	F	Medley	13
Sub08	M	Medley	16
Sub09	F	Free Style	13
Sub10	M	Breaststroke	11
Sub11	F	Free Style	16
Sub12	M	Free Style /Medley	14
Sub13	F	Breaststroke	16
Sub14	M	Backstroke	15
Sub15	M	Butterfly	11
Sub16	F	Backstroke	13

Table S3. Technical grade standard for swimmers (Men)

**Men**	International Master		Master		National First Class		National Second Class		National Third Class	
	50M Pool	25M Pool	50M Pool	25M Pool	50M Pool	25M Pool	50M Pool	25M Pool	50M Pool	25M Pool
50M Free Style	21.95	20.95	23.28	22.28	24.50	23.50	27.50	26.50	34.50	33.50
100M Free Style	48.39	46.89	51.50	50.00	55.50	54.00	1:05.00	1:03.50	1:22.00	1:20.50
200M Free Style	1:46.88	1:43.38	1:51.55	1:48.05	2:03.00	1:59.50	2:23.00	2:19.50	2:56.00	2:52.50
400M Free Style	3:47.79	3:40.79	3:58.60	3:51.60	4:21.00	4:14.00	5:06.00	4:59.00	6:16.00	6:11.50
800M Free Style	8:00.09	7:46.09	8:24.00	8:10.00	9:02.00	8:48.00	10:32.00	10:18.00	13:12.00	12:58.00
1500M Free Style	15:12.08	14:47.08	16:00.30	15:35.30	17:20.00	16:54.00	20:15.00	19:50.00	24:45.00	24:20.00
50M Backstroke	25.24	24.24	27.43	26.43	30.50	29.50	35.50	34.50	43.00	42.00
100M Backstroke	53.92	52.92	58.45	57.45	1:04.00	1:03.00	1:14.00	1:13.00	1:30.00	1:29.00
200M Backstroke	1:57.90	1:55.90	2:06.45	2:04.45	2:18.00	2:16.00	2:41.00	2:39.00	3:16.00	3:13.00
50M Breaststroke	27.47	26.47	28.75	27.75	32.50	31.50	37.00	36.00	44.00	43.00
100M Breaststroke	1:00.23	58.23	1:03.80	1:01.80	1:11.00	1:09.00	1:20.00	1:18.00	1:34.00	1:32.00
200M Breaststroke	2:11.75	2:07.75	2:21.90	2:17.90	2:35.00	2:31.00	2:54.00	2:50.00	3:23.00	3:19.00
50M Butterfly	23.51	22.51	24.89	23.89	27.00	26.00	32.50	31.50	41.50	40.50
100M Butterfly	51.92	50.42	55.45	53.95	1:00.00	58.50	1:11.00	1:09.50	1:29.00	1:27.50
200M Butterfly	1:56.59	1:53.59	2:02.70	1:59.70	2:14.00	2:11.00	2:38.00	2:35.00	3:18.00	3:15.00
200M Medley	1:59.83	1:56.83	2:08.20	2:05.20	2:19.00	2:16.00	2:40.00	2:37.00	3:15.00	3:12.00
400M Medley	4:18.05	4:12.05	4:31.20	4:25.20	4:58.30	4:52.00	5:31.00	5:25.00	6:56.00	6:50.00

Table S4. Technical grade standard for swimmers (Women)

**Women**	International Master		Master		National First Class		National Second Class		National Third Class	
	50M Pool	25M Pool	50M Pool	25M Pool	50M Pool	25M Pool	50M Pool	25M Pool	50M Pool	25M Pool
50M Free Style	24.90	23.90	25.85	24.85	27.20	26.20	31.50	30.50	38.50	37.50
100M Free Style	54.35	52.85	56.30	54.80	1:02.50	1:01.00	1:13.00	1:11.00	1:34.00	1:33.00
200M Free Style	1:57.84	1:54.34	2:01.20	1:57.70	2:15.00	2:11.50	2:39.00	2:35.50	3:23.00	3:19.50
400M Free Style	4:09.17	4:02.17	4:15.80	4:08.80	4:44.00	4:37.00	5:46.00	5:39.00	7:06.00	6:59.00
800M Free Style	8:33.95	8:19.95	8:53.40	8:39.40	9:42.00	9:28.00	12:02.00	11:48.00	15:02.00	14:48.00
1500M Free Style	16:37.40	16:12.46	17:14.00	16:49.00	18:35.00	18:10.00	23:45.00	23:20.00	27:45.00	27:20.00
50M Backstroke	28.61	27.61	30.55	29.55	33.00	32.00	38.50	37.50	46.50	45.50
100M Backstroke	1:00.69	59.69	1:04.30	1:03.30	1:09.00	1:08.00	1:21.00	1:20.00	1:41.00	1:40.00
200M Backstroke	2:10.10	2:08.10	2:18.30	2:16.30	2:29.50	2:27.00	2:53.00	2:51.00	3:38.50	3:36.50
50M Breaststroke	31.23	30.23	31.70	30.70	36.00	35.00	41.00	40.00	48.00	47.00
100M Breaststroke	1:07.41	1:05.41	1:10.75	1:08.75	1:18.00	1:16.00	1:29.00	1:27.00	1:44.00	1:42.00
200M Breaststroke	2:25.19	2:21.19	2:36.60	2:32.60	2:51.00	2:47.00	3:13.00	3:09.00	3:48.00	3:44.00
50M Butterfly	26.47	25.47	27.50	26.50	30.50	29.50	36.50	35.50	45.50	44.50
100M Butterfly	58.11	56.61	1:00.50	59.00	1:08.00	1:06.50	1:20.00	1:18.50	1:39.00	1:37.50
200M Butterfly	2:08.75	2:05.75	2:14.20	2:11.20	2:25.00	2:22.00	2:54.50	2:51.50	3:38.00	3:35.00
200M Medley	2:12.84	2:09.84	2:18.40	2:15.40	2:30.00	2:27.00	2:58.00	2:55.00	3:48.00	3:45.00
400M Medley	4:41.61	4:35.61	4:56.80	4:50.80	5:18.00	5:12.00	6:21.00	6:15.00	8:06.00	8:00.00

Supplementary References

Bartolomeo, P., and Seidel Malkinson, T. (2019). Hemispheric lateralization of attention processes in the human brain. *Curr Opin Psychol* 29, 90-96. doi: 10.1016/j.copsyc.2018.12.023.

Harmon-Jones, E., and Allen, J.J. (1997). Behavioral activation sensitivity and resting frontal EEG asymmetry: covariation of putative indicators related to risk for mood disorders. *J Abnorm Psychol* 106(1), 159-163. doi: 10.1037//0021-843x.106.1.159.

Harmon-Jones, E., and Allen, J.J. (1998). Anger and frontal brain activity: EEG asymmetry consistent with approach motivation despite negative affective valence. *J Pers Soc Psychol* 74(5), 1310-1316. doi: 10.1037//0022-3514.74.5.1310.

LeMay, M. (1977). Asymmetries of the skull and handedness. Phrenology revisited. *J Neurol Sci* 32(2), 243-253. doi: 10.1016/0022-510x(77)90239-8.

Maki-Marttunen, V., Villarreal, M., and Leiguarda, R.C. (2014). Lateralization of brain activity during motor planning of proximal and distal gestures. *Behav Brain Res* 272, 226-237. doi: 10.1016/j.bbr.2014.06.055.

Neubauer, S., Gunz, P., Scott, N.A., Hublin, J.J., and Mitteroecker, P. (2020). Evolution of brain lateralization: A shared hominid pattern of endocranial asymmetry is much more variable in humans than in great apes. *Science Advances* 6(7). doi: ARTN eaax9935

10.1126/sciadv.aax9935.

Nielsen, J.A., Zielinski, B.A., Ferguson, M.A., Lainhart, J.E., and Anderson, J.S. (2013). An Evaluation of the Left-Brain vs. Right-Brain Hypothesis with Resting State Functional Connectivity Magnetic Resonance Imaging. *Plos One* 8(8). doi: ARTN e71275

10.1371/journal.pone.0071275.

Smit, D.J.A., Posthuma, D., Boomsma, D.I., and De Geus, E.J.C. (2007). The relation between frontal EEG asymmetry and the risk for anxiety and depression. *Biological Psychology* 74(1), 26-33.

Tzourio-Mazoyer, N., Josse, G., Crivello, F., and Mazoyer, B. (2004). Interindividual variability in the hemispheric organization for speech. *Neuroimage* 21(1), 422-435. doi: 10.1016/j.neuroimage.2003.08.032.

Zheng, G.X., Qi, X.Y., Li, Y.Z., Zhang, W., and Yu, Y.G. (2018). A Comparative Study of Standardized Infinity Reference and Average Reference for EEG of Three Typical Brain States. *Frontiers in Neuroscience* 12. doi: ARTN 158

10.3389/fnins.2018.00158.

I.I. Goncharova, D.J. McFarland, T.M. Vaughan, J.R. Wolpaw. EMG contamination of EEG: spectral and topographical characteristic. (2003) Clinical Neurophysiology 114: 1580-1593

German Gomez-Herrero. Automatic Artifact Removal (AAR) toolbox v1.3 (Release 09.12.2007) for MATLAB (2007)
